# Enzymatic Oxidation of Carbohydrate Byproducts for Use in Formation of Chitosan Hydrogels

**DOI:** 10.1002/cbic.202500559

**Published:** 2025-11-10

**Authors:** Owen Mototsune, Yutong Zhang, Durgesh Kavishvar, Arun Ramchandran, Michele C. Loewen, Emma Master

**Affiliations:** ^1^ Department of Chemical Engineering and Applied Chemistry University of Toronto 200 College Street Toronto ON M5S 3E5 Canada; ^2^ Department of Chemistry and Biomolecular Sciences University of Ottawa 30 Marie Curie Ottawa ON K1N 6N5 Canada; ^3^ Department of Bioproducts and Biosystems Aalto University Kemistintie 1 FI‐00076 Espoo Finland

**Keywords:** chitosan, crosslinker, galactose oxidase, hydrogel, pyranose dehydrogenase, xylooligosaccharides

## Abstract

Chitosan hydrogels are used in diverse applications ranging from pharmaceuticals and biomedical materials to food and agriculture. This study introduces a biology‐inspired approach to create fully bio‐based hydrogels by combining chitosan with bio‐based di/polycarbonyl crosslinkers produced through the enzymatic oxidation of carbohydrates. Two such crosslinkers, Ox‐XOS and Ox‐Lac, were synthesized by oxidizing carbohydrates: Ox‐XOS was produced by oxidizing xylooligosaccharides (XOS) with pyranose dehydrogenase from *Agaricus bisporus* (*Ab*PDH1), and Ox‐Lac was produced by oxidizing lactose (Lac) with galactose oxidase from *Fusarium graminearum* (*Fgr*GalOx). The efficacy of enzymatic oxidation of lactose and XOS was analyzed using liquid chromatography and mass spectroscopy, showing high degrees of oxidation, and carbonyl groups were confirmed using ATR‐FTIR and ^1^H NMR. Compared with unmodified XOS, Ox‐XOS showed a lower reaction temperature towards hexamethylenediamine by differential scanning calorimetry and demonstrated stronger gel formation ability with polyallylamine and chitosan. Rheological measurements showed O(10)–$O(10^{3})$ increases in the storage moduli (G′) of chitosan hydrogels formed with Ox‐Lac and Ox‐XOS compared with unmodified lactose and XOS, indicating considerable increases in the hydrogels’ resistance to deformation. These findings demonstrate the potential of enzymatically oxidized carbohydrates as crosslinkers to enhance chitosan hydrogels with potential utility in both high‐value and large‐volume sectors.

## Graphical abstract

Carbohydrates, i.e., xylooligosaccharides derived from corn cobs and lactose derived from dairy byproducts, were selectively oxidized using fungal oxidoreductases *Ab*PDH1 (pyranose dehydrogenase from *Agaricus bisporus*) and *Fgr*GalOx (galactose oxidase from *Fusarium graminearum*). The resulting oxidized carbohydrates then crosslink chitosan, a crustacean shell biopolymer, to produce bio‐based hydrogels for agricultural or biomedical applications.

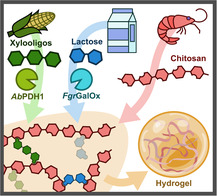

## 1 Introduction

Carbonyl crosslinkers, such as formaldehyde, glyoxal, and glutaraldehyde, are important chemicals used in many large‐scale applications, including adhesives and textiles, with production volumes in millions of tonnes per year.^[^ ^1^, ^2^ ^–^ ^3^ ^]^ However, such synthetic crosslinkers are derived from fossil fuels and pose significant adverse effects to environmental and human health, making them prime candidates for replacement with benign bio‐based alternatives.^[^ ^4^, ^5^, ^6^ ^–^ ^7^ ^]^ This process is currently underway, with regulations in force limiting formaldehyde use in resins in composite wood materials.^[^ ^8^, ^9^ ^–^ ^10^ ^]^

Hemicelluloses are an abundant yet underused component of plant cell walls (i.e., lignocellulosic biomass), which can be extracted from wood chips before pulping and from agricultural residuals of biofuel production (e.g., corn fiber, sugarcane bagasse). In plants, hemicelluloses are thought to connect the cellulose and lignin components of plant cell walls while imparting flexibility to the lignocellulose network, making them compelling starting materials for making alternatives to synthetic crosslinkers.^[^ ^11^ ^,^ ^12^ ^]^ Xylans represent a major hemicellulose type and principally include glucuronoxylans produced by hardwoods (dicots) and glucuronoarabinoxylans produced by grasses and cereals (commelinid monocots). Xylans comprise of 50–200 repeating units of D‐xylopyranose linked together by *β*‐(1→4) glycosidic bonds and decorated with other mono‐ or oligosaccharide side groups.^[^ ^11^, ^12^ ^–^ ^13^ ^]^ Xylans can be extracted from lignocellulosic biomass and fragmented to oligosaccharides by a combination of hot water pretreatment and enzymatic hydrolysis.^[^ ^14^, ^15^ ^–^ ^16^ ^]^ These xylooligosaccharides (XOSs) have been studied mainly for feed, food, and nutraceutical applications;^[^ ^14^, ^15^ ^–^ ^16^ ^]^ their potential material applications remain largely unexplored.

Besides hemicelluloses, lactose (*β*‐D‐galactopyranosyl‐(1→4)‐D‐glucose) is a byproduct of dairy processing and another underused and abundant carbohydrate.^[^ ^17^ ^]^ In 2021–2024, ≈500 000 t of lactose was produced annually in the USA.^[^ ^18^ ^]^ Currently, lactose is used as an additive in food and as a feedstock to make lactobionic acid (antioxidant), lactitol (low calorie sweetener), and lactulose (prebiotic).^[^ ^17^ ^]^ Due to its low sweetness and the prevalence of lactose intolerance, lactose represents a suitable carbon source for uses other than food.^[^ ^19^ ^]^

To render XOSs and lactose suitable for use as bio‐based carbonyl crosslinkers, carbonyl functionalities must be introduced at multiple positions on the molecules. Although chemical methods exist to modify carbohydrates with carbonyls, each presents recognized limitations. Periodate oxidation, converts vicinal diols in carbohydrates to two aldehydes,^[^ ^20^, ^21^ ^–^ ^22^ ^]^ including in monosaccharides (i.e., glucose),^[^ ^23^ ^]^ oligosaccharides (e.g., sucrose, cyclodextrin),^[^ ^21^ ^,^ ^24^ ^]^ and polysaccharides (e.g., alginate, amylose, pectin, chitosan, cellulose, xyloglucan, and xylan).^[^ ^20^, ^21^ ^–^ ^22^ ^,^ ^25^ ^,^ ^26^ ^]^ However, periodate oxidation requires harmful reagents and can lead to depolymerization and increase the polydispersity of carbohydrate products.^[^ ^20^, ^21^ ^–^ ^22^ ^,^ ^27^ ^]^ Sodium hypochlorite (NaOCl) oxidations have been used to oxidize hydroxypropyl cellulose and hydroxypropyl dextran to introduce ketone groups at the oligo(hydroxypropyl) terminal secondary hydroxyl positions.^[^ ^20^ ^]^ This approach requires the hydroxypropylation of carbohydrates before sodium hypochlorite oxidation, which typically occurs at high temperatures and pressures and requires the use of petroleum‐based propylene oxide.^[^ ^28^ ^]^ Esterifications, using 4‐formylbenzoate or levulinate groups, or acetoacetylation, using a diketene, are common strategies for grafting carbonyl functional groups onto carbohydrates.^[^ ^20^ ^]^ These esterification pathways require synthetic reagents and organic solvents, undergo side reactions leading to low yields and degrees of substitution, and result in products with low tolerance to heat.^[^ ^20^ ^]^ None of these pathways are especially attractive from a green chemistry perspective.

**Scheme 1:**
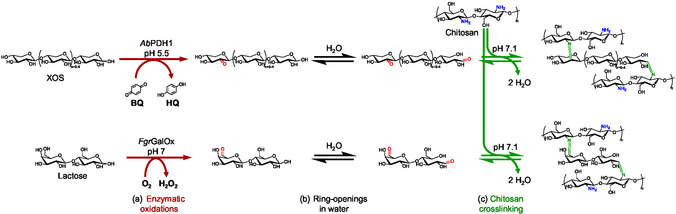
Enzymatic oxidations of XOS by *Ab*PDH1 to produce a C‐2 ketone and lactose by *Fgr*GalOx to produce a C‐6 aldehyde group a) ring openings in water to convert C‐1 anomeric hemiacetal to a C‐1 aldehyde b) and reaction between chitosan and Ox‐XOS or Ox‐Lac to from imine crosslinks.

Alternatively, enzymatic oxidations of carbohydrates can be performed at ambient temperatures, in water at physiological pH, and without reducing the degree of polymerization of the substrates.^[^ ^29^, ^30^, ^31^, ^32^ ^–^ ^33^ ^]^ Carbohydrate‐active enzymes (CAZymes) belonging to auxiliary activity (AA) families AA3 and AA5 are especially well suited for introducing carbonyl in XOS and lactose, respectively. For example, pyranose dehydrogenases (PDH, AA3_2, EC 1.1.99.29) have been reported to oxidize the C‐2 or C‐3 secondary hydroxyls to the corresponding ketones, while reducing quinones (i.e., benzoquinone, 2,6‐dichlorophenolinophenol) or organometallic ions (i.e., ferrocenium) (**Scheme** **1**a).^[^ ^30^ ^,^ ^34^ ^]^ Pyranose dehydrogenase from *Agaricus bisporus* (*Ab*PDH1) was shown to oxidize both the terminal subunits of xylooligosaccharides, producing dicarbonyl carbohydrates with high conversions: In proof‐of‐concept work by Karppi et  al., *Ab*PDH1 was able to reach 81% conversion of xylotriose (*β*‐D‐Xyl*p*‐(1→4)‐*β*‐D‐Xyl*p*‐(1→4)‐D‐Xyl*p*) and 48% conversion of xylotetraose (*β*‐D‐Xyl*p*‐(1→4)‐*β*‐D‐Xyl*p*‐(1→4)‐*β*‐D‐Xyl*p*‐(1→4)‐D‐Xyl*p*) to their respective double‐oxidized counterparts.^[^ ^30^ ^]^ *Ab*PDH1 is a ≈75 kDa flavin‐dependent oxidoreductase, containing a flavin adenine dinucleotide (FAD) binding domain with a covalently‐linked FAD cofactor. So far, PDHs have been studied for applications in biosensors for various sugars, several biofuel cell configurations, and the biocatalysis of tagatose (prebiotic and low‐calorie sweetener) and lactulose (laxative).^[^ ^34^ ^]^

Besides pyranose dehydrogenases, carbonyl groups have also been introduced to carbohydrates using galactose oxidase (GalOx, AA5_2, EC 1.1.3.9), which oxidizes the C‐6 primary hydroxyl of D‐galactose to the corresponding aldehyde (i.e., galacto‐hexodialdose), while reducing molecular oxygen to hydrogen peroxide (Scheme 1a). GalOx is capable of acting on galactose or terminal galactopyranosyl subunits of oligosaccharides (i.e., lactose) and polysaccharides (i.e., galactoglucomannan).^[^ ^35^ ^]^ Galactose oxidase from *Fusarium graminearum* (*Fgr*GalOx) is a well‐studied single‐copper metalloenzyme with a molecular weight ≈65 kDa. *Fgr*GalOx has been used in several applications, including biosensors, monomer production (e.g., diformylfuran and furan dicarboxylic acid), the synthesis of pharmaceuticals (e.g., naftifine, islatravir, L‐3‐aminopiperidine, L‐3‐aminoazepane), the modification of galactose‐containing polysaccharides for gels and functional materials for food and medical applications, and with other enzymatic or chemical process to chemically modify mono‐ and polysaccharides (amination, carboxylation, and allylation).^[^ ^13^ ^,^ ^36^ ^,^ ^37^ ^]^

In addition to the carbonyl groups introduced by *Ab*PDH1 or *Fgr*GalOx, XOS and lactose are reducing sugars containing an anomeric C‐1 hemiacetal that exists in equilibrium with the aldehyde form (Scheme 1b). Carbohydrates exist in the closed ring form over 99% of the time, but as the aldehyde form is consumed, the equilibrium drives more free aldehyde, allowing the C‐1 aldehyde to act as a second crosslinking point.^[^ ^38^ ^,^ ^39^ ^]^

We previously demonstrated the application of *Ab*PDH1 and *Fgr*GalOx to produce di/polycarbonyl crosslinkers from galactose and lactose and their use to form hydrogels with polyallylamine (PAA).^[^ ^40^ ^]^ In this work, *Ab*PDH1 was used to oxidize xylose and a mixture of xylooligosaccharides (XOS) derived from corn cob xylan to produce Ox‐Xyl and Ox‐XOS, respectively, and *Fgr*GalOx was used to oxidize lactose to produce Ox‐Lac. Ox‐XOS (i.e., XOS oxidized by *Ab*PDH1) and Ox‐Lac (i.e., lactose oxidized by *Fgr*GalOx) were then tested for their ability to produce fully bio‐based hydrogels by crosslinking chitosan, the most abundant natural source of polyamines, through dynamic covalent imine crosslinks (Scheme 1c).^[^ ^41^ ^]^ Fully bio‐based chitosan hydrogels are of interest in slow release fertilizers for agriculture^[^ ^42^ ^,^ ^43^ ^]^ and for human health applications in tissue engineering and wound healing,^[^ ^44^ ^,^ ^45^ ^]^ where chitosan's biocompatibility, biodegradability, and antimicrobial properties could be particularly useful. This work adds to the growing body of research into enzyme‐assisted chitosan crosslinking, which includes the use of tyrosinases and transglutaminases in generating chitosan hydrogels and films.^[^ ^46^, ^47^, ^48^ ^–^ ^49^ ^]^

## 2 Experimental Section

### 2.1 Materials

Chitosan from shrimp shells (product number 417,963, reported 75% deacetylation, reported MW 190–375 kDa, reported Brookfield viscosity at 1% > 200 cP) and 10% w/w polyallylamine in water (product number 479,144, reported MW ≈65 kDa) were purchased from Millipore Sigma. Xylooligosaccharides (XOS) derived from corn cobs were donated by Shandong Longlive Biotechnology Co., Ltd (product number XOS95P), batch 95P20221017). Standards of xylose and xylobiose (Millipore Sigma) and xylotriose, xylotetraose, xylopentaose, and xylohexaose (Megazyme) were used to determine the weight percent of each xylooligosaccharide in XOS. Lactose, xylose, galactose, glucosamine hydrochloride, benzoquinone, acetic acid, imidazole, sodium hydroxide, sodium chloride, ABTS, hydroquinone, and deuterium oxide were also purchased from Millipore Sigma. Glycerol, potassium phosphate monobasic, potassium phosphate dibasic, sodium phosphate monobasic, sodium phosphate dibasic, yeast extract, peptones, and biotin were purchased from BioShop Canada. Catalase (E.C. 1.11.1.6, from bovine liver, Millipore Sigma), laccase (E.C. 1.10.3.2, Novozym 51,003, Novozymes), and horseradish peroxidase (E.C. 1.11.1.7, from horseradish, Millipore Sigma) were used.

### 2.2 Enzyme Production and Purification

Enzymes were produced as previously reported.^[^ ^33^ ^]^ In brief, *Ab*PDH1 and *Fgr*GalOx were produced heterologously in *Komagataella* *phaffii* strain KM71H. YPD plates were streaked and incubated at 30 °C for 3 days. From the plates, 5 × 1 L volumes of BMGY in 4 L flasks were inoculated and grown at 30 °C, shaking at 220 rpm for 1 day. Cells were then collected by centrifugation at 3000 × g and resuspended in 5 × 200 mL of BMMY (1% methanol) and then pooled into 1 L induction culture in a 4 L shake flask. Induction was conducted over 4 days at 10 °C, shaking at 220 rpm. Methanol was replenished to 1% methanol (i.e., adding 10 mL) each day.

To purify the recombinant proteins, the cultivation pH was adjusted to 7.5 with sodium hydroxide, cells were pelleted, and the supernatant was decanted. The crude supernatant was then filtered through a 0.45 μm capsule membrane filter (Cytiva), adjusted to contain 10 mM imidazole and then loaded onto a 5 mL HisTrap FF Crude column (Cytiva) and eluted with an increasing imidazole gradient from 10 to 300 mM. Fractions containing the target protein were pooled and buffer exchanged into 50 mM pH 7.5 sodium/phosphate or 50 mM pH 5.5 ammonium/acetate buffer, for *Fgr*GalOx, and *Ab*PDH1, respectively, using 10 kDa centrifugal filtration systems. Activity was measured as previously reported using the ABTS‐HRP oxidase activity assay for *Fgr*GalOx and the BQ dehydrogenase activity assay for *Ab*PDH1.^[^ ^40^ ^]^ One *Fgr*GalOx activity unit (U) is equal to 1 μmol of H_2_O_2_ produced per minute (=2 μmol of ABTS oxidized to 2 μmol of ABTS•+ per minute). One *Ab*PDH1 activity unit (U) is equal to 1 μmol of hydroquinone produced per minute.

### 2.3 Preparation of Ox‐Lac and Ox‐XOS Crosslinkers

Ox‐Lac synthesis reactions were performed in water and comprised 5.4 g of lactose (150 mM), 580 U mL^−1^ (1.1 mg mL^−1^) *Fgr*GalOx, 81 U mL^−1^ HRP (units based on activity reported by manufacturer on ABTS with H_2_O_2_) and 1600 U mL^−1^ catalase (units based on activity reported by manufacturer on H_2_O_2_).^[^ ^40^ ^]^ The final reaction volume was 20 mL in 125 mL Erlenmeyer flasks; five replicate reactions were prepared and incubated at 200 rpm and 30 °C for 6 h. A control flask without *Fgr*GalOx (Ctrl‐Lac) and a control flask with only lactose (Std‐Lac) were also prepared (**Table** **1**).

**Table 1:** Reagent concentrations in *Ab*PDH1 and *Fgr*GalOx respective enzyme oxidations of XOS and lactose.

Crosslinker	XOS [mg mL^−1^]	BQ [mM]	Laccase [U mL^−1^]	*Ab*PDH1 [U mL^−1^]		Crosslinker	Lactose [mM]	Catalase [U mL^−1^]	HRP [U mL^−1^]	*Fgr*GalOx [U mL^−1^]
Ox‐XOS	10	1	3	1.25		Ox‐Lac	150	1600	81	580
Ctrl‐XOS	10	1	3	0		Ctrl‐Lac	150	1600	81	0
Std‐XOS	10	0	0	0		Std‐Lac	150	0	0	0

Crosslinker	Xylose [mM]	BQ [mM]	Laccase [U mL^−1^]	*Ab*PDH1 [U mL^−1^]	pH 5.5 buffer [mM]					
Ox‐XOS	17	1	3	1.25	2					
Ctrl‐XOS	17	1	3	0	2					
Std‐XOS	17	0	0	0	0					

Ox‐XOS synthesis reactions were performed at pH 5.5, where *Ab*PDH1 is most active, and comprised 0.14 mM acetic acid, 3.0 g of XOS (10 mg mL^−1^), 1.25 U mL^−1^ (39 μg mL^−1^) *Ab*PDH1, 1 mM benzoquinone (electron acceptor), and 3 U mL^−1^ laccase (based on activity measured on hydroquinone).^[^ ^40^ ^]^ Six replicates of 50 mL reactions in 250 mL Erlenmeyer flasks were prepared and incubated at 200 rpm and 30 °C for 24 h. To avoid buffer impurities in the final product, a 2 M NaOH solution was used to keep the pH between 5 and 6, reaching an end NaOH concentration of 7.2 mM. Control conditions included all reaction components except *Ab*PDH1 (Ctrl‐XOS) and a flask comprising only XOS (Std‐XOS) (Table 1).

Ox‐Xyl synthesis reactions were performed in 2 mM pH 5.5 sodium acetate buffer and comprised 0.5 g of xylose (17 mM), 1.25 U mL^−1^ (39 μg mL^−1^) *Ab*PDH1, 1 mM benzoquinone (electron acceptor), and 3 U mL^−1^ laccase (based on activity on hydroquinone). Four replicates of 50 mL reactions in 250 mL Erlenmeyer flasks were prepared and incubated at 200 rpm and 30 °C for 24 h. A control flask without any *Ab*PDH1 (Ctrl‐Xyl) and a control flask with only xylose (Std‐Xyl) were also prepared (Table 1).

Reactions were stopped by cooling on ice and removing enzymes using 10 kDa Jumbosep centrifugal filters (Cytiva) to prevent enzymes from continuing to act on other reaction components in subsequent experiments or to participate in reactions. Activity assays confirmed effective removal of enzymes with 10 kDa cutoff filters, with <0.1% remaining *Fgr*GalOx in Ox‐Lac and 1% remaining *Ab*PDH1 and 5% remaining laccase in Ox‐XOS. Filtrates were then freeze dried and stored at ‐20 °C. Control (i.e., Ctrl‐XOS, Ctrl‐Xyl, and Ctrl‐Lac) and standard (i.e., Std‐XOS, Std‐Xyl, and Std‐Lac) were treated to the same purification procedure to yield solids.

### 2.4 HPAEC‐PAD to Quantify Xylooligosaccharide Mixture Composition

To quantify the composition of the mixture of xylooligosaccharides from corn cobs, equimolar amounts of X1–X6 were dissolved in water and diluted to produce a standard curve spanning 0.005–0.3 mM. XOS was dissolved at 10, 30, 100, and 300 μg mL^−1^. Aliquots (12.5 μL) were injected into a CarboPac PA200 guard column (3 × 50 mm) and analytical column (3 × 250 mm) at 0.5 mL min^−1^ with an increasing concentration of sodium acetate and sodium hydroxide over 50 min. For the first 25 min, the samples were injected with 100% Eluent C (1 mM sodium hydroxide). Then, over the next 20 min, Eluent A (200 mM sodium hydroxide and 1 M sodium acetate) and Eluent B (200 mM sodium hydroxide) increased in a linear gradient to 50% each, while Eluent C decreased to 0%. Eluent A and B were then dropped to 0% and Eluent C was set to 100% for 15 min. Data were analyzed using the Chromeleon Chromatography Data System version 7.2 (Thermo Fisher Scientific). Standard curves were constructed from 12 duplicate concentrations, with data points fit by a nonlinear exponential growth to plateau model: Y = Y_M_‐(Y_M_‐Y_0_)*exp(‐k^*^x), where Y is the integral of the chromatography peak (nC*min), Y_M_ is the maximum integral of the chromatography peak (nC*min), k is the rate constant (unitless), and x is the analyte concentration (mol L^−1^). XOS integrals (*n *= 2–4) were used to calculate molarities, which were then used to calculate mass concentrations, and thus mass percent of XOS.

### 2.5 HPAEC‐PAD and HILIC‐ESI‐MS to Verify the Enzymatic Oxidation of Carbohydrates

Samples were taken at 0, 2, 5, 20, and 26 h for Ox‐XOS reactions and controls; at 0, 1, 3, 4.5, 6, and 6.1 h for Ox‐Lac reactions and controls; and 0, 4, 16, and 24 h for Ox‐Xyl samples and controls. Samples were filtered by 10 kDa centrifuge filters to remove enzymes and diluted to suitable concentrations (≈0.5 mM) for analysis by high‐performance anion‐exchange chromatography with pulsed amperometric detection (HPAEC‐PAD). To ensure all samples were assessed in the linear range of the PAD detector, multiple endpoint measurements were taken. For Ox‐XOS samples and controls, endpoints were measured at 0.057, 0.171, and 0.285 mg mL^−1^, and other timepoints were measured only once at 0.285 mg mL^−1^. For Ox‐Lac samples and controls, endpoints were injected once at 0.2, 0.6, and 1 mM, and other timepoints were measured only once at 1 mM. For Ox‐Xyl samples and controls, endpoints were measured 0.1, 0.3, and 0.5 mM, and other timepoints were measured only at 0.5 mM. Injections (12.5 μL) were loaded through a CarboPac PA1 guard column (2 × 50 mm) and analytical column (2 × 250 mm) at 0.25 mL min^−1^ with an increasing concentration of sodium acetate and sodium hydroxide over 50 min. The column was equilibrated with 50% Eluent B (200 mM sodium hydroxide) and 50% Eluent C (1 mM sodium hydroxide). After sample injection, Eluent A (200 mM sodium hydroxide in 1 M sodium acetate) was increased 0%–10% over 35 min while decreasing both Eluent B and C to 45%. Eluent A was then increased from 10% to 20% over 10 min, further reducing Eluent B and C contributions to 40% each. Lastly, Eluent A was increased to 50% over 5 min while decreasing both Eluent B and C to 25%. Data were analyzed using the Chromeleon Chromatography Data System version 7.2 (Thermo Fisher Scientific). Conversions were calculated as substrate depletions, comparing oxidized carbohydrates to unmodified standards and control reactions.

Hydrophobic interaction liquid chromatography coupled with electrospray ionization mass spectrometry (HILIC‐ESI‐MS) was used to verify depletion of substrates and accumulation of products. Samples taken periodically during oxidation reactions (see previous paragraph for sampling times) were filtered through 10 kDa centrifuge filters to remove enzymes, diluted to suitable concentrations (≈0.5 mM) in Milli‐Q water and then filtered through 0.22 μm filters. Aliquots (10 μL) were then injected at 0.4 mL min^−1^ into a Thermo Scientific Ultimate 3000 UHPLC system with a Waters Acquity BEH HILIC (3.0 mm × 150 mm, 1.7 μm) equipped with a guard column at 40 °C. After equilibration for 1 min at 50% Eluent A (acetonitrile) and 50% Eluent B (20 mM ammonium acetate and 20 mM ammonium hydroxide in water), the samples were eluted using a linear gradient to 100% Eluent B over 1 min, holding at 100 % Eluent B for 9 min, reducing to 50% Eluent B over 0.5 min and then holding at 50% Eluent B for 3.5 min. A Thermo Scientific Q‐Exactive equipped with a HESI‐II probe (spray voltage, 3.5 kV; capillary temperature:, 320 °C) was used for mass spectroscopy. Full MS was performed over a m/z range of 70−1000 with a resolution of 140,000 and AGC target of 3× 10^6^ in both positive and negative polarity mode. Data‐dependent MS^2^ was performed with an isolation window of 0.4 m z^−1^, with a resolution of 17,500, and an AGC target of 1 × 10^5^. The logarithm (1og_10_) of all peak integral data values (counts/s) for oxidized products, controls, and standards were converted to a color based on a color scale of red (low) to green (high).

### 2.6 Differential Scanning Calorimetry to Compare the Reactivity of Ox‐Lac and Ox‐XOS Crosslinkers

Differential scanning calorimetry (DSC) was conducted as previously described.^[^ ^40^ ^]^ In brief, 1.32 M crosslinker and 1.32 M hexamethylene diamine (HMDA) were suspended in water, and 5–20 mg of that suspension was loaded into high pressure capsules. A molar mass of 360.31 g mol^−1^ was used for lactose crosslinkers calculations, a molar mass of 282.24 g mol^−1^ (the molar mass of xylobiose) was used for XOS crosslinkers, and 150.13 g mol^−1^ was used for xylose crosslinkers. A TA Instruments Q2000 DSC system was used to equilibrate the capsule at 5 °C for 10 min and then to increase the temperature of the samples to 200 °C at 5 °C min^−1^. Raw data were analyzed using TA Universal Analysis version 4.5 A. Smoothing was applied over ranges of 1−2 °C. Enthalpies of reaction were calculated by integrating peaks with a sigmoidal tangent baseline. Peak temperature is defined as the temperature at which the curve is furthest from the baseline. Onset/offset points are defined as the intersection of the baseline tangent line with the tangent after/before the on/offset.

### 2.7 Gelation of Polyallylamine and Chitosan Using Ox‐Lac and Ox‐XOS Crosslinkers

As previously reported, 100 μL of 10% polyallylamine (PAA) solutions were mixed with 18 μmol of crosslinkers for 100 mg mL^−1^ PAA experiments and 100 μL of 5% PAA solutions were mixed with 9.0 μmol of crosslinkers in 200 μL PCR tubes.^[^ ^40^ ^]^ Since XOS is a mixture with multiple chemical species (e.g., xylose, xylobiose, xylotriose, xylotetraose, xylopentaose, xylohexaose), the molar mass of XOS was approximated as the molar mass of xylobiose (i.e., 282.24 g mol^−1^), so 18 μmol of Ox‐XOS is 5.1 mg, and 9.0 μmol of Ox‐XOS is 2.5 mg. Glutaraldehyde was used as a positive control and gluconic acid was used as a negative control.

A stock solution of 30 mg mL^−1^ chitosan was prepared in 0.26 M acetic acid, stirring overnight to dissolve the chitosan. The stock was diluted with water and 0.5 M sodium phosphate dibasic (Na_2_HPO_4_) to make 12 chitosan solutions with Na_2_HPO_4_ concentrations 0, 50, 75, 100, 125, 150, 175, 200, 225, 250, 275, and 300 mM, resulting in pH values 4.35–7.43. The final chitosan and acetic acid concentrations were 8 mg mL^−1^ and 70 mM, respectively. To avoid chitosan aggregation or precipitation, chitosan was added to the vessel first, with water and then 0.5 M Na_2_HPO_4_ added after. The mixture was immediately vortexed to yield a translucent and homogenous solution.

Different amounts of crosslinker (40, 12.5, and 4 mM) were added to 7.4 mg mL^−1^ chitosan solutions, with the assumption that crosslinkers with better performance could successfully induce gelling at a lower crosslinker concentration. For the sake of Ox‐XOS calculations, the molar mass of XOS is approximated as that of xylobiose (i.e., 282.24 g mol^−1^), so a 40 mM Ox‐XOS solution had a concentration of 11.3 mg mL^−1^. Lactose crosslinkers used a molar mass of 360.31 g mol^−1^, so a 40 mM Ox‐Lac solution had a concentration of 14.4 mg mL^−1^. Hydrogel mixtures with 40 mM crosslinker were prepared by adding 92 μL of the chitosan solution containing 8 mg mL^−1^ chitosan, 150 mM Na_2_HPO_4_, and 70 mM acetic acid and 8 μL of 0.5 M crosslinker to a 200 μL PCR tube, yielding a final chitosan concentration of 7.4 mg mL^−1^ (46 mM glucosaminyl repeating unit), 61 mM acetic acid, and 138 mM Na_2_HPO_4_. For 12.5 mM crosslinker mixtures, 92 μL of 8 mg mL^−1^ chitosan solution, 2.5 μL of 0.5 M crosslinker, and 5.5 μL of water were used. For 4 mM crosslinker, 92 μL of 8 mg mL^−1^ chitosan solution and 8 μL of 0.05 M crosslinker was used. Ox‐XOS and Ox‐Lac were compared against controls and standards from the enzymatic oxidations (i.e., Ctrl‐XOS, Std‐XOS, Ctrl‐Lac, Std‐Lac) as well as negative (i.e., lactobionic acid) and positive controls (i.e., glutaraldehyde). Visual detection of gelation was recorded for qualitative assessment of crosslinker performance.

### 2.8 ATR‐FTIR

Attenuated total reflectance Fourier‐transform infrared (ATR‐FTIR) spectroscopy using a Bruker Alpha ATR‐FTIR was used to initially verify the enzymatic introduction of aldehyde (1731 cm^−1^) and ketone (1735 cm^−1^) groups in the carbohydrate substrates were observed.^[^ ^40^ ^]^ Solid samples (≈5 mg) were loaded directly onto the instrument for analysis. Following cross‐linking reactions with polyamines, ATR‐FTIR was used to verify the depletion of the carbonyl peaks and with primary amine peak (1577 cm^−1^). Chitosan hydrogels used in the analysis were freeze dried and analyzed, based on a previously reported protocol.^[^ ^24^ ^]^ Spectra were generated using data from 24 scans from 400 to 4000 cm^−1^ and analyzed using Bruker OPUS version 7.2.

### 2.9 Nuclear Magnetic Resonance Spectroscopy

Crosslinker solutions were added to a solution of glucosamine hydrochloride in deuterium oxide (D_2_O) to achieve the same amine concentration (i.e., 46 mM glucosamine, 9.9 mg mL^−1^) and same crosslinker concentration (i.e., 40 mM crosslinker) as in chitosan hydrogel formation experiments. Ctrl‐XOS, Std‐XOS, Ctrl‐Lac, and Std‐Lac were also tested for reactivity with glucosamine. Acetic acid (61 mM) and sodium phosphate dibasic (Na_2_HPO_4_, 138 mM) were also added to match concentrations in hydrogel experiments. Reactions were pipetted into 3 mm NMR tubes and measured after 2 and 48 h of reaction time at room temperature.

1D ^1^H and all 2D NMR spectra were acquired at 25 °C using an Agilent DD2 spectrometer equipped with a 5 mm variable temperature HCN Cold Probe (n(^1^H) = 699.803 MHz, n(^13^C) = 175.984 MHz; Agilent Technologies, Santa Clara, CA, USA). Samples were locked, tuned, and shimmed prior to data acquisition, and spectra were recorded using vendor‐supplied pulse sequences.

1D ^1^H measurements were carried out using the PROTON pulse sequence as supplied in the VnmrJ software package. Spectra were acquired over a 11,160.7 Hz spectral window using 100,446 points (4.5s acquisition time), a 5s relaxation delay, 32 dB receiver gain, 2 steady state scans, and 16 transients.

2D COSY‐45 spectra were carried out using a gCOSY pulse sequence as supplied in the VnmrJ software package. Spectra were acquired over a 8064.5 Hz spectral window using 2048 points in F_2_ and 8064.5 Hz spectral window using 256 increments in F_1_. Spectra were acquired with a 1.0s d1 delay, 32 dB receiver gain, four steady state scans, and one transient.

2D ^1^H/^13^C multiplicity‐edited HSQC spectra were carried out using a gHSQCAD pulse sequence as supplied in the VnmrJ software package. Spectra were acquired over an 8064.5 Hz spectral window using 2048 points in F_2_ and 40,465.4 Hz spectral window using 128 increments in F_1_. Spectra were acquired a 140 Hz j1xh delay, a 1.0s d1 delay, 54 dB receiver gain, four steady state scans, two transients, and ^1^H inverse‐gated decoupling using a WURST40 decoupling waveform.

2D ^1^H/^13^C HMBC spectra were carried out using a gHMBCAD pulse sequence as supplied in the VnmrJ software package. Spectra were acquired over a 6648.9 Hz spectral window using 2048 points in F_2_ and 39,604.0 Hz spectral window using 128 increments in F_1_. Spectra were acquired with a 140 Hz j1xh delay, an 8 Hz jnxh delay, a 1.0s d1 delay, 54 dB receiver gain, eight steady state scans, and four transients.

NMR processing was carried out using MestreNova software (v 14.3.1‐31739 Santiago de Compostela, Research S.L., Spain). All NMR spectra were Fourier transformed, phased, and baseline corrected using a third order Bernstein polynomial function for all other spectra. ^1^H and ^13^C chemical shifts were referenced relative to residual solvent peaks (D_2_O, *δ*(^1^H) = 4.80 ppm) or indirectly to the lock signal. Twofold linear prediction and zero‐filling were applied in the F_1_ dimension for all 2D NMR spectra along with appropriate modifications to the default apodization provided by the NMR processing software. Additional t_1_ noise reduction was optionally applied to reduce t_1_‐ridges when necessary.

^1^H spectra were reference to the D_2_O peak at 4.80 ppm. Peak integrals were normalized to 1.00 for H1 signals for both anomers (i.e., anomeric H*α*1 and H*β*1 signals) of XOS: H*α*1 (*δ *= 5.22 ppm, d, *J *= 3.7 Hz, integral = 0.36) and H*β*1 (*δ *= 4.62 ppm, d, *J *= 7.8 Hz, integral = 0.65) and for glucosamine: H*α*1 (*δ *= 5.47 ppm, d, *J *= 3.6 Hz, integral = 0.60) and H*β*1 (*δ *= 4.94 ppm, d, *J *= 8.4 Hz, integral = 0.41). For lactose, glycosidic H1’ (*δ *= 4.49 ppm, d, *J *= 7.8 Hz, integral = 1.00) integrals were normalized to 1.00. Peaks were assigned based on complementary analysis from 1D and 2D experiments, as well as previously reported data.^[^ ^38^ ^,^ ^50^, ^51^, ^52^, ^53^, ^54^, ^55^ ^–^ ^56^ ^]^

### 2.10 Rheological Measurements

A chitosan solution containing 8 mg mL^−1^ chitosan, 70 mM acetic acid, and 150 mM Na_2_HPO_4_ was produced at pH 7.1 using the protocol described to produce chitosan gels. A 460 μL sample of the chitosan solution and 40 μL of 0.5 M carbohydrate crosslinker (i.e., Ox‐XOS, Ctrl‐XOS, Std‐XOS, Ox‐Lac, Ctrl‐Lac, and Std‐Lac) were mixed to produce a solution with 7.4 mg mL^−1^ chitosan, 138 mM Na_2_HPO_4_, and 40 mM crosslinker and then stored for 7 days at room temperature in closed 10 mL scintillation vials. An additional control for the Ox‐XOS sample was tested, called BQ‐XOS, which contained 40 mM XOS and 1.1 mM BQ, which is the same XOS:BQ molar ratio as used in Ox‐XOS and Ctrl‐XOS reactions.

Rheological experiments were conducted using a TA Instruments DHR‐3 rheometer, equipped with a 60 mm sandblasted parallel plate geometry. The measurement gap was set to 500 µm, and all experiments were performed at a controlled temperature of 25 °C. Two types of oscillatory rheology studies were carried out: small amplitude oscillatory shear (SAOS) to assess the linear viscoelastic behavior, and large amplitude oscillatory shear (LAOS) to explore nonlinear viscoelastic properties.

In the SAOS regime, frequency sweeps were conducted over a range of 0.1–100 rad s^−1^, with a constant strain amplitude of 1%, during which the storage modulus ($G^{\prime }$) and loss modulus ($G^{\prime \prime }$) were recorded. For LAOS, strain amplitudes were varied between 0.1% and 1000%, while the frequency of oscillation was fixed at 10 rad s^−1^, with $G^{\prime }$ and $G^{\prime \prime }$ also being recorded. The choice of 10 rad s^−1^ for LAOS was made because, at lower frequencies such as 0.1 rad s^−1^, the rheometer did not produce a detectable response for dilute or liquid‐like samples, necessitating a higher frequency to obtain measurable rheological data.

In the temporal study, Ox‐XOS and chitosan solutions were mixed near the rheometer, with the timing of the experiment initiated at the moment of mixing. The sample was then placed on the rheometer, and the parallel plate geometry was lowered to the operating gap of 500 µm. Rheological measurements began 5 min after mixing to monitor the time‐dependent evolution of the gel's viscoelastic properties. $G^{\prime }$ and $G^{\prime \prime }$ were recorded as a function of time, using an oscillation frequency of 1 rad s^−1^ and a strain amplitude of 1%. This procedure allowed us to capture the dynamic gelation process of the sample.

## 3 Results and Discussion

### 3.1 Crosslinker Production

HPAEC‐PAD measurements were first used to determine the composition of the mixture of xylooligosaccharide from corn cobs (XOS), using standards of xylose (X1), xylobiose (X2), xylotriose (X3), xylotetraose (X4), xylopentaose (X5), and xylohexaose (X6) (**Table** **2**). By weight, XOS contains mostly X2 (47%), X3 (29%), and X4 (6.2%) which together account for 82% of the total weight. Almost no X1 (0.7%) is present. As standards for xylooligosaccharides larger than X6 were not available (e.g., X7, X8), their presence at low quantities cannot be ruled out. Overall, X1–X6 account for about 90% of the total XOS mass, with the remaining 10% assumed to be larger linear xylooligosaccharides or other impurities (e.g., xylooligosaccharides with arabinose, ferulic acid, or methylglucuronic acid modifications).

**Table 2:** XOS composition as determined by HPAEC‐PAD based on standards of xylooligosaccharides. *M*_i _= molar mass of mixture component, *i*. *W*_i _= mass fraction of mixture component, *i*. *X*_i _= number fraction of mixture component, *i*. *σ *= standard deviation, based on *n* measurements. The sum of $X_{i}\;*\;M_{i}$ terms gives the number average molecular weight (*M*_n_), and the sum of $\frac{W_{i}}{\sum W_{i}}\;*\;M_{i}$ terms gives the weight average molecular weight (*M*_w_).

Xylooligosaccharide	M_i_ [g mol^−1^]	μmol_i_/g_total_	W_i_	X_i_	*σ*	n	$X_{i}{\;*\;M}_{i}$ [g mol^−1^]	$\frac{W_{i}}{\sum W_{i}}{\;*\;M}_{i}$ [g mol^−1^]
Xylose (X1)	150.13	17	0.3%	1%	0.01%	2	1.0	0.4
Xylobiose (X2)	282.24	1657	47%	64%	1.7%	4	181.2	146.6
Xylotriose (X3)	414.36	691	29%	27%	2.0%	4	110.9	131.7
Xylotetraose (X4)	546.47	114	6.2%	4%	0.6%	4	24.1	37.7
Xylopentaose (X5)	678.6	13	0.9%	0%	0.1%	2	3.3	6.5
Xylohexaose (X6)a)	810.7	90	7.3%a)	3%	0.6%	4	28.3	65.8
Sum		2581	90%	100%	5%		348.7	388.7

a) As standards for xylooligosaccharides larger than X6 were not available (e.g., X7, X8), their coelution with X6 cannot be ruled out.

**Figure 1:**
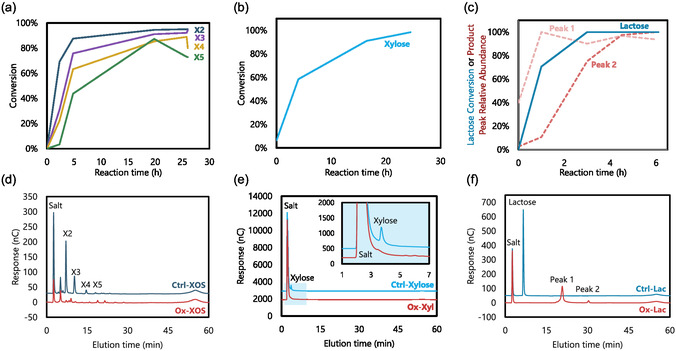
HPAEC‐PAD analysis showing depletion of substrates as enzymatic oxidations progress. Oxidation of XOS by *Ab*PDH1, showing conversion of xylooligosaccharides X2, X3, X4, and X5 a) Oxidation of xylose by *Ab*PDH1 b) and oxidation of lactose by *Fgr*GalOx c) End point HPAEC‐PAD chromatograms comparing no‐enzyme controls to *Ab*PDH1 oxidation of XOS d) and xylose e) and *Fgr*GalOx oxidation of lactose f) Ctrl‐XOS, Ctrl‐Xyl, and Ctrl‐Lac are the no‐enzyme controls for their corresponding enzyme‐oxidized carbohydrate counterparts Ox‐XOS, Ox‐Xyl, and Ox‐Lac, respectively.

After enzymatic oxidations of carbohydrates were conducted to produce Ox‐XOS, Ox‐Xyl, and Ox‐Lac, HPAEC‐PAD measurements were again used to determine the conversion. For Ox‐XOS (i.e., xylooligosaccharides oxidized by *Ab*PDH1), 96% ± 1% xylobiose (X2), 95% ± % 0.4% xylotriose (X3), 82% ±2% xylotetraose (X4), and 69% ± 9% xylopentaose (X5) were consumed, as measured by HPAEC‐PAD (**Figure** **1**a). With X2, X3, and X4 accounting for 82% of the total XOS by weight (Table 2), this result confirms a high degree of oxidation of the major XOS components. Xylose and xylohexaose peaks in the XOS were observed but were too low to permit accurate quantification.

Meanwhile, HPAEC‐PAD measurements of Ox‐Xyl and Ox‐Lac showed high conversions: respectively, xylose consumption increased for the entire 24 h reaction time to a final conversion of 97% ± 2, and lactose depletion reached 100% after 3 h (Figure 1b,c). Two new peaks were identified in the Ox‐Lac chromatogram, which are not observed in the chromatogram of the no‐enzyme lactose control (Ctrl‐Lac) and lactose alone (Std‐Lac): the first peak reached a plateau after 1 h, while the second peak continued to increase for the full 6 h reaction time (Figure 1c). The first peak is likely the single‐oxidized lacto‐hexodialdose, and the second peak is likely the double‐oxidized lacturonic acid, although these assignments could not be confirmed as no standards were available (Figure 1f).

Similarly, HILIC‐ESI‐ToF‐MS showed depletion of substrates and formation of products for both *Ab*PDH1 oxidation of XOS and Xyl, and *Fgr*GalOx oxidation of lactose (Figure S1, Supporting Information). The accumulation of double‐ and triple‐oxidized products over time was detected in Ox‐XOS samples. While the conversion of xylose and X6 in Ox‐XOS was not accurately quantified by HPAEC‐PAD, mass spectrometry confirmed their conversion by *Ab*PDH1 (Figure S1a, Supporting Information). Mass spectrometry analysis of Ox‐Lac was consistent with the HPAEC analysis, with lactose conversion and single‐oxidized lactose reaching a plateau at 3 h, while double‐oxidized lactose increase for the whole reaction time (Figure S1b, Supporting Information).

FTIR showed the introduction of ketone peaks at 1735 and 1603 cm^−1^ in Ox‐XOS and an aldehyde peak at 1731 cm^−1^ in Ox‐Lac (Figure S2, Supporting Information), supporting the evidence of oxidation shown by HPAEC and mass spectrometry.

^1^H NMR further verified the oxidation of XOS by *Ab*PDH1 (Figure S3, Supporting Information). Despite being unable to directly detect ketones (as they have no C–H bonds), the oxidation of C‐2 protons was observed, with the H*α*2 (*δ *= 3.61–3.57 ppm, m) and H*β*2, H2’ and H2’’; (*δ *= 3.32–3.26 ppm, m) signals decreasing in intensity relative to other peaks. Signals for H*α*1 (*δ *= 5.22 ppm, d, *J *= 3.7 Hz) and H*β*1 (*δ*=4.62 ppm, d, *J *= 7.8 Hz) protons were also observed to have decreased to 0, indicating the oxidation of the C‐1 to the corresponding acid, but could also have been shifted due to their proximity to the oxidized C‐2 hydroxyls. Protons connected to glycosidic bonds H1’ and H1’’; (*δ *= 4.48–4.52 ppm, m) were also observed to have shifted from their original chemical shifts in response to the *Ab*PDH1‐catalyzed reaction.

^1^H NMR additionally showed strong evidence of oxidation of lactose by *Fgr*GalOx (Figure S4, Supporting Information). Signals from galactosyl H6’ protons (H*α*6^′^: *δ *= 3.82 ppm, dd, *J *= 3.2, 0.8 Hz; *δ* = 3.78 ppm, dd, *J *= 3.7, 0.9 Hz; H*β*6^′^: *δ *= 3.83 ppm, d, *J *= 4.0 Hz; *δ* = 3.80 ppm, dd, *J *= 3.9, 0.9 Hz) were reduced to 0 and replaced with a small aldehyde peak H6’’A (*δ *= 9.61 ppm, s) after the *Fgr*GalOx‐catalyzed reaction. A larger geminal diol peak H6’’ (*δ *= 5.19 ppm; ddd, *J *= 7.4, 3.2, 0.6 Hz) was also observed, which is commonly observed for carbohydrate aldehydes dissolved in D_2_O.^[^ ^35^ ^,^ ^56^ ^]^ Protons H5’ (*δ *= 3.77–3.75 ppm, m) and H4’ (*δ *= 3.96 ppm, d, *J *= 3.4 Hz) also shifted in response to the oxidation at C‐6, with protons H5’’ (*δ *= 3.50, dd, *J *= 7.4, 0.9 Hz) and H4’’ (*δ *= 4.12 ppm, dd, *J *= 3.4, 1.1 Hz) respectively replacing them.

### 3.2 Reactivity Comparison Using DSC

Following confirmation of enzymatic oxidations, the peak reaction temperatures of oxidized carbohydrates (i.e., Ox‐XOS and Ox‐Xyl) with hexamethylenediamine (HMDA) were compared using DSC against unmodified carbohydrate controls, in which *Ab*PDH1 was not included in the enzymatic oxidation reaction mixture but were otherwise prepared in the same way (i.e., Ctrl‐XOS for Ox‐XOS and Ctrl‐Xyl for Ox‐Xyl) and standards (i.e., Std‐XOS for Ox‐XOS and Std‐Xyl for Ox‐Xyl). DSC is typically used to observe physical changes in response to changes in temperature; it can also be used to observe chemical changes. By sealing reagents (i.e., HMDA and an oxidized carbohydrate) in a capsule and increasing temperature, heat flow variations can indicate exothermic/endothermic chemical reactions occurring. Decreases in reaction temperature are then used as an indication of increased readiness to react and thus higher reactivity.

**Figure 2:**
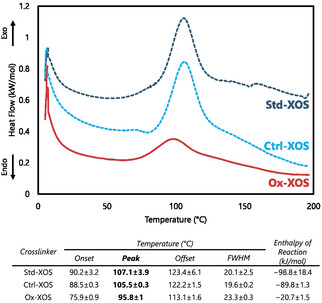
DSC showing decrease in reaction temperature for Ox‐XOS toward HMDA compared to Ctrl‐XOS and Std‐XOS. *n *= 3 for Std‐XOS, Ox‐XOS; *n *= 2 for Ctrl‐XOS.

Although HMDA is not a bio‐based chemical, it was selected as a benchmark for imine forming reactions as it has been used previously in similar studies. For example, in a previous publication, we reported the reaction temperatures of HMDA toward lactose‐based crosslinkers (i.e., Ox‐Lac and unmodified lactose) showed no appreciable difference between one another.^[^ ^40^ ^]^ Contrastingly, the peak reaction temperature of HMDA with *Fgr*GalOx‐oxidized galactose (*Fgr*GalOx‐Gal) was 34 °C less than the peak reaction temperature of HMDA with unmodified galactose.^[^ ^40^ ^]^ Herein, the DSC analyses revealed a decrease in peak reaction temperature of ≈10 °C for Ox‐XOS with HMDA compared with reactions of HMDA with Ctrl‐XOS and Std‐XOS (**Figure** **2**).

Ox‐Xyl was also tested for reactivity with HMDA, which indicated that after oxidation by *Ab*PDH1, no reaction occurred between Ox‐Xyl and HMDA (Figure S5, Supporting Information). This observation is likely explained by the oxidation of the C‐1 forming xylonic acid (Scheme S2, Supporting Information). Carboxylic acids (e.g., xylonic acid) react with amines in an acid–base proton exchange rather than the covalent bond forming reactions between carbonyls at temperatures below 200 °C; accordingly, reaction detection by DSC was not expected.^[^ ^40^ ^]^

### 3.3 Gelation of Polyallylamine and Chitosan Solutions

**Scheme 2:**
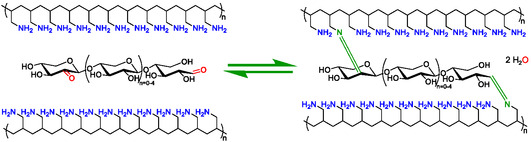
Reaction between PAA and Ox‐XOS to form imine crosslinks.

Given the reactivity of both Ox‐XOS and Ox‐Lac with HMDA, the oxidized carbohydrates were compared in terms of their ability to crosslink polyamines, forming visible hydrogels (**Scheme** **2**). In previous work, Ox‐Lac was shown to induce gelation of 100 mg mL^−1^ polyallylamine (PAA) in 4 min and 50 mg mL^−1^ PAA in 15 min at room temperature.^[^ ^40^ ^]^ In this study, Ox‐XOS induced the gelation of 100 mg mL^−1^ PAA in 15 min and induced the gelation of 50 mg mL^−1^ PAA in 2 days at room temperature (**Table** **3**, Figure S6, Supporting Information).

**Table 3:** Hydrogel formation time for PAA and chitosan solutions with different amounts of Ox‐XOS, Ox‐Xyl, and Ox‐Lac crosslinkers, with controls and standards. PAA experiments were conducted at room temperature and pH ≈ 12. Pictures of gels are shown in Figures S6 and S7, Supporting Information. Chitosan experiments were conducted at room temperature, 138 mM Na_2_HPO_4_, 63 mM acetic acid, and pH ≈ 7.

Crosslinker		PAA		Chitosan		
		1.75 M RUb) [100 mg mL^−1^] PAA	0.88 M RUb) [50 mg mL^−1^] PAA	46 mM RUb) [7.4 mg mL^−1^] chitosan		
		175 mM crosslinker	88 mM crosslinker	40 mM crosslinker	12.5 mM crosslinker	4 mM crosslinker
XOS crosslinkers	Ox‐XOS	15 min	2 days	20 min	1.5 h	No gelling
	Ctrl‐XOS	3 days	No gelling	19 h	No gelling	No gelling
	Std‐XOS	3 days	No gelling	No gelling	No gelling	No gelling
Lactose crosslinkersa)	Ox‐Lac	4 mina)	10 mina)	19 h	2 days	7 days
	Ctrl‐Lac	n.d.	n.d.	No gelling	No gelling	No gelling
	Std‐Lac	6 daysa)	No gellinga)	No gelling	No gelling	No gelling
Xylose crosslinkers	Ox‐Xyl	2 days	No gelling	n.d.	n.d.	n.d.
	Ctrl‐Xyl	7 days	No gelling	n.d.	n.d.	n.d.
	Std‐Xyl	7 days	No gelling	n.d.	n.d.	n.d.
Controls	Xylonolactone (‐ctrl)	No gelling	No gelling	n.d.	n.d.	n.d.
	Lactobionic acid (‐ctrl)a)	No gellinga)	No gellinga)	No gelling	No gelling	No gelling
	Glutaraldehyde (+ctrl)	Immediately	Immediately	Immediately	Immediately	Immediately

a) Tested and reported previously in Mototsune et al. Biomacromolecules 2024:

b) RU = repeating unit:

n.d. not determined.

Reactions with 100 mg mL^−1^ PAA and Ctrl‐XOS and Std‐XOS gelled in 3 days at room temperature, likely through the Maillard reaction, which typically occurs at elevated temperatures, but can occur at slower rates at room temperature.^[^ ^57^, ^58^ ^–^ ^59^ ^]^ The Maillard reaction is a complex scheme of reactions that can produce a dicarbonyl intermediate, which can then react with two amines to crosslink polymers through imine bonds, with a variety of other products subsequently formed.^[^ ^60^ ^]^ This non‐enzymatic crosslinking was observed with previously reported crosslinkers.^[^ ^40^ ^]^

Next, the ability of Ox‐XOS and Ox‐Lac to form hydrogels was tested using the bio‐based polyamine, chitosan. Before conducting the crosslinking experiments, the impact of pH on chitosan gel formation was tested by varying the amount of Na_2_HPO_4_ to set the pH from 4.4 to 7.4. For both Ox‐XOS and Ox‐Lac, a relatively narrow pH range of 7.0–7.2 was identified for optimal gel formation (Table S1, Supporting Information). It was therefore decided that a pH 7.1 Na_2_HPO_4_ solution (138 mM) would be used for the remaining experiments. Multivalent ions such as phosphate can participate in ionic crosslinking with chitosan; however, since all reactions contained the same concentration of phosphate, the impact to hydrogel formation would be the same across all samples, and thus no differences should be observed due to ionic interactions with phosphate.

It is important to note here that although Ox‐Lac and Ox‐XOS are both generated by enzymatic oxidations of carbohydrates, they do not contain the same functional groups and therefore are not expected to be equally reactive. *Fgr*GalOx introduces an aldehyde, while *Ab*PDH1 introduces a ketone, and in general, aldehydes are more reactive than ketones in reactions with amines; therefore, Ox‐Lac is expected to be more reactive than Ox‐XOS. The formation of PAA gels was indeed quickest when using Ox‐Lac; however, the formation of chitosan‐derived gels was quickest when using Ox‐XOS (Table 3). For example, in chitosan reactions comprising 40 mM crosslinker, Ox‐XOS formed hydrogels in 20 min while Ox‐Lac formed hydrogels in 19 h.

Clearly, when considering peak reaction temperature and gelation time, no single crosslinker is the best for every polymer. Instead, the crosslinker and the polymer to be crosslinked must been chosen together to optimize hydrogel formation for the desired gelling time.^[^ ^61^ ^]^ The pK_a_ value of the polyamine and the pH of the reaction affect crosslinking. The pK_a_ for PAA is ≈ 9.3,^[^ ^62^ ^]^ while for chitosan, the value is ≈6.5.^[^ ^63^ ^,^ ^64^ ^]^ The pH of PAA reactions was ≈10, while it was much lower ≈7.1 for chitosan reactions. Crosslinker reactivity could also be pH dependent, which would favor one crosslinker over another depending on the pH of the reaction. The relative density or concentration of amine groups could also be an important factor. While both PAA and chitosan contain one amine group per repeating unit, they differ significantly in their structure: PAA has a much smaller repeating unit (‐C_3_H_7_N‐) than chitosan (‐C_6_H_11_O_4_N‐), with amine groups closer together in PAA than in chitosan. Additionally, PAA experiments were conducted at relatively high concentrations of PAA (100 mg mL^−1^ = 1.75 M repeating unit), while chitosan concentrations (7.4 mg mL^−1^ = 46 mM repeating unit) were far lower; at lower concentrations of PAA, no gels formed, while at higher concentrations of chitosan, viscosity was too high to properly investigate gelation. At higher concentrations, the ability to span longer distances may be less necessary, which could be why Ox‐Lac, despite being a disaccharide, was more quickly able to crosslink PAA solutions to form gels with its reactive aldehyde groups. Meanwhile, chitosan solutions, which were tested at much lower concentrations, potentially require a longer crosslinker to effectively span the distances between polymer chains in a more dilute solution, favoring Ox‐XOS. XOS is made up of ≈36% xylotriose, xylotetraose, and xylopentaose. It is possible that for the PAA experiments, the reactivity of the carbonyl groups was more important to faster gel formation, while for the chitosan experiments, the larger size of crosslinkers was more important, although it is likely the combination of many factors. More data is clearly needed to effectively predict speed of hydrogel formation.

At 40 mM, Ctrl‐XOS, which lacked *Ab*PDH1 but included all other reaction components, formed a chitosan gel in 19 h, while Std‐XOS did not form a gel even after 7 days. This observation suggests that the other reaction components (i.e., laccase or benzoquinone) have an effect on gel formation. Laccase activity measurements confirmed 99% removal of laccase from Ctrl‐XOS after the reaction, so it is unlikely that the laccase directly participated in the hydrogel formation. Laccases can create radicals on ferulic acid groups, which are present in corn xylan, from which XOS is derived. The ferulic acid radicals can subsequently recombine to link two xylooligosaccharide molecules forming a feruloyl dimer (Scheme S3, Supporting Information).^[^ ^13^ ^,^ ^65^ ^,^ ^66^ ^]^ The dimerization of feruloylated xylooligosaccharides could allow them to act as crosslinkers through the C‐1 anomeric hemiacetal/aldehyde bond (Scheme S3, Supporting Information). Despite this possibility, when investigated using LC‐MS, no change in the abundance of feruloylated xylooligosaccharides was detected in Ctrl‐XOS over time or between Ctrl‐XOS and Std‐XOS samples; in other words, in the presence of laccase but without *Ab*PDH1 (Ctrl‐XOS), no changes were observed in the feruloylated xylooligosaccharides measurements, providing no evidence that the laccase acts directly on feruloylated xylooligosaccharides in a measurable way (Figure S8, Supporting Information).

Benzoquinone is also present in Ctrl‐XOS, and unlike laccase, it is too small (M = 108.10 g mol^−1^) to be filtered out by the 10 kDa filter. Benzoquinone and other related quinones have been demonstrated to react with chitosan amine groups to form imine or secondary amine bonds (Scheme S4, Supporting Information)^[^ ^63^ ^,^ ^67^ ^]^ and shown to form hydrogels with chitosan through such crosslinks.^[^ ^68^ ^]^ The hypothesis that benzoquinone is responsible for the hydrogel formation in Ctrl‐XOS was investigated by rheological measurements, as described in the next section of the results.

**Figure 3:**
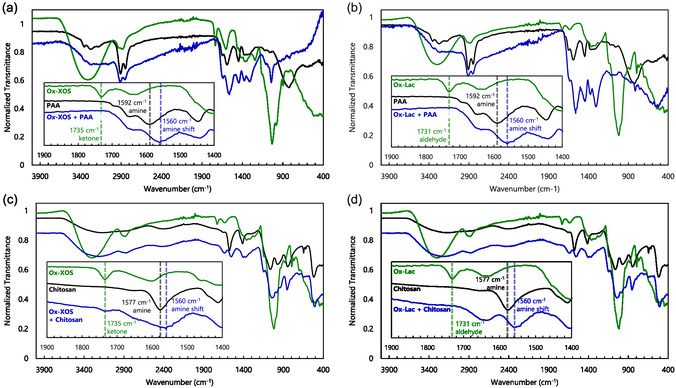
Crosslinking of PAA a,b) or chitosan c,d) by Ox‐XOS (a,c) or Ox‐Lac (b,d), followed by ATR‐FTIR. Carbonyl (ketone for Ox‐XOS [a,c] and aldehyde for Ox‐Lac [b,d]) peaks ≈1730 cm^−1^ deplete, amine peak in PAA at 1592 cm^−1^, or in chitosan at 1577 cm^−1^ shift to a peak at ≈1560 cm^−1^.

PAA and chitosan hydrogels were frozen and freeze dried and then analyzed by ATR‐FTIR (**Figure** **3**). In all crosslinked hydrogel samples, carbonyl peaks (ketone peak at 1735 cm^−1^ for Ox‐XOS and aldehyde peak at 1731 cm^−1^ for Ox‐Lac) clearly decreased, indicating they were consumed in reactions with polyamines. Amine peaks at 1592 cm^−1^ for PAA and at 1577 cm^−1^ for chitosan were shifted to slightly lower wavenumbers ≈1560 cm^−1^, although this shift is also observed in the reactions with controls (i.e., Ctrl‐XOS and Ctrl‐Lac) and standards (i.e., Std‐XOS and Std‐Lac) making it difficult to attribute this shift to imine formation (Figure S9, Supporting Information). Moreover, imine peaks are typically observed at wavenumbers around 1600–1640 cm^−1^, which overlap with other peaks observed in the reagents, making it difficult to confirm imine formation by FTIR.

**Figure 4:**
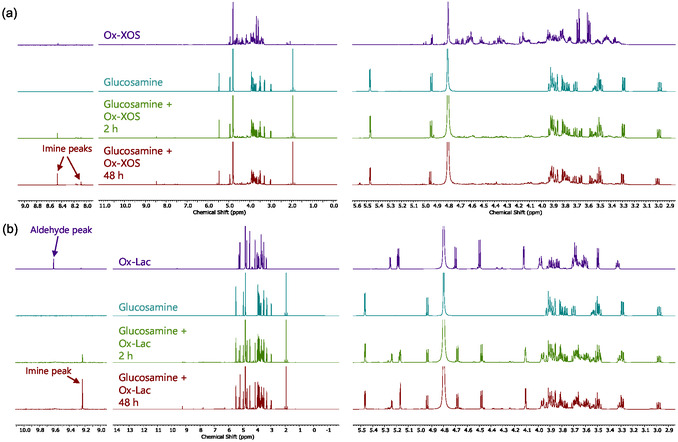
^1^H NMR (700 MHz, d_2_o) showing imine formation in glucosamine reactions with Ox‐XOS a) and Ox‐Lac b) Detailed 1D and 2D spectra for Ox‐XOS, Ox‐Lac, and GluN can be found in Figures S13, S14, and S17–S20, Supporting Information.

To confirm imine bond formation, ^1^H NMR was also used. Due to the necessity of deuterated solvents for ^1^H NMR and chitosan's lower solubility in deuterium oxide (D_2_O) compared to water, chitosan could not be analyzed by ^1^H NMR to adequate sensitivity. Instead, glucosamine, the monomer of chitosan, was reacted with Ox‐XOS and Ox‐Lac under the same conditions as chitosan in the hydrogel formation experiments. NMR experiments showed time‐dependent increases in the formation of imine bonds in both reactions with Ox‐XOS and Ox‐Lac with glucosamine, the repeating unit of chitosan (**Figure** **4**). In Ox‐XOS reactions with glucosamine, two imine peaks appeared in the expected range for imines (*δ *= 8.47, s; *δ *= 8.20–8.05 ppm, m). Likewise, for reactions with Ox‐Lac, an imine peak appeared (*δ *= 9.235 ppm, s) and the aldehyde peak (*δ *= 9.61 ppm, s) disappeared. The geminal diol H6’’; (*δ *= 5.19 ppm, dd) signal does not change significantly after GluN is added to Ox‐Lac. In both samples with Ox‐XOS and Ox‐Lac, these imine peak integrals were relatively small (0.06 and 0.03, respectively, for Ox‐XOS and 0.05 for Ox‐Lac compared to glucosamine protons with integrals of 1.00), suggesting only about 5%–9% of glucosamine and crosslinker (2.3–4.1 mM) participate in imine forming reactions. Despite the low conversion in these measurements, it is likely that reactions with glucosamine in D_2_O behave slightly differently than reactions with chitosan in water, and most important, the ^1^H NMR measurements confirmed the formation of imine bonds between the glucosamine and carbonyl crosslinkers. Controls and standards (i.e., Ctrl‐XOS, Std‐XOS, Ctrl‐Lac, and Std‐Lac) did not show evidence of imine peak formation after 48 h or reaction (Figure 4). By combining FTIR, which confirms carbonyl consumption, and ^1^H NMR, which confirms imine bond formation, evidence is provided for imine crosslinking occurring in the chitosan hydrogels.

### 3.4 Rheological Measurements on Various Chitosan Gels

**Figure 5:**
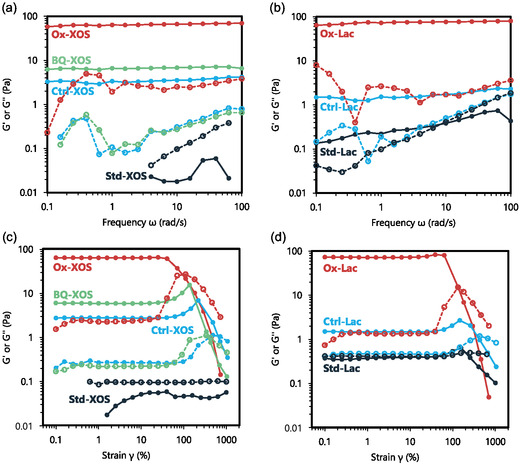
Storage modulus (G′), shown in filled circles, and loss modulus ($G^{\prime \prime }$), shown with empty circles, measurements for chitosan gels with ox‐XOS a,b) and Ox‐Lac c,d) in which oscillation frequency *ω* (a,c) and shear strain amplitude *γ* (b,d) are varied. Ox‐XOS is compared against BQ‐XOS (control with no *Ab*PDH1 or laccase), Ctrl‐XOS (control with no *Ab*PDH1), and Std‐XOS (control with no *Ab*PDH1, laccase, or BQ). Ox‐Lac is compared against Ctrl‐Lac (control with no *Fgr*GalOx) and Std‐XOS (control with no *Fgr*GalOx, catalase, or HRP). Experiments were conducted at room temperature, 138 mM Na_2_HPO_4_, 63 mM acetic acid, and pH ≈ 7.

To expand on qualitative observations of the impact of the enzyme‐oxidized crosslinkers on chitosan hydrogel formation, rheological measurements were used quantify the viscoelastic properties of the resulting chitosan hydrogels and to quantify gelation time. The viscoelastic response of chitosan gels was characterized by measuring the storage modulus ($G^{\prime }$) and loss modulus ($G^{\prime \prime }$), where $G^{\prime }$ quantifies a material's elastic, solid‐like behavior, while $G^{\prime \prime }$ reflects the viscous, liquid‐like properties.^[^ ^69^ ^]^ Two types of rheological experiments for crosslinked chitosan gels were conducted: (1) small amplitude oscillatory shear (SAOS, **Figure** **5**a,b) to probe linear viscoelastic behavior when gel structure is intact and (2) large amplitude oscillatory shear (LAOS, Figure 5c,d) to probe nonlinear viscoelastic behavior when gel structure breaks down. LAOS measurements can be used to calculate the yield stress or yield point which is a key rheological parameter defined as the point at which a gel's viscoelastic properties transition from solid‐like behavior to liquid‐like behavior.^[^ ^69^ ^,^ ^70^ ^]^

The chitosan solution containing unmodified Std‐XOS had $G^{\prime }$ measurements that range from $O(10^{-2})$ to $O(10^{-1})$ Pa, while the chitosan hydrogel with Ox‐XOS exhibited a $G^{\prime }$ three orders of magnitude greater ($O(10^{2})$ Pa) (Figure 3a). The Std‐XOS containing chitosan solution had values of $G^{\prime \prime }>G^{\prime }$, indicating a liquid‐like behavior, while the Ox‐XOS chitosan hydrogels had $G^{\prime }>G^{\prime \prime }$, demonstrating gel‐like behavior. These results underscore the substantial impact of the *Ab*PDH1 oxidation on the viscoelastic properties of chitosan and match the qualitative observations of chitosan gel formation presented above that showed chitosan with Ox‐XOS formed gels in just 20 min, while Std‐XOS did not form gels with chitosan.

Similarly, the chitosan hydrogel made using Ox‐Lac displayed a $G^{\prime }$of $O(10^{2})$ Pa, which is three orders of magnitude greater than that of chitosan with unmodified lactose (Std‐Lac), where $G^{\prime }$ is of $O(10^{-1})$ Pa (Figure 3b). The Ox‐Lac chitosan hydrogel had a $G^{\prime }$ approximately $\left(10^{2}\right)$ greater than $G^{\prime \prime }$, indicating predominantly solid‐like behavior. Meanwhile, the Std‐Lac containing chitosan sample displayed $G^{\prime }>G"$ at frequencies below 6rad/s, and $G^{\prime }<G^{\prime \prime }$ at higher frequencies, suggesting a transition from solid‐like behaviors at low frequencies to liquid‐like behavior at higher frequencies. Notably, hydrogels with $G^{\prime }$ values of $O(10^{2})$ Pa have been used in experimental formulations for slow release fertilizers^[^ ^71^ ^]^ for stabilizing biopesticides,^[^ ^72^ ^]^ and in flame prevention materials,^[^ ^73^ ^]^ which could be potential applications for the Ox‐XOS and Ox‐Lac crosslinked chitosan hydrogels generated herein.

As mentioned in the previous section, it was hypothesized that residual BQ in Ctrl‐XOS could participate in reactions with chitosan, causing gelation in reactions with Ctrl‐XOS and chitosan. To investigate the impact of BQ on chitosan hydrogel properties, a mixture of BQ, XOS, and chitosan (BQ‐XOS) was prepared. Chitosan mixtures with Ctrl‐XOS (containing BQ, laccase, and XOS) and BQ‐XOS (containing BQ and XOS) exhibited similar $G^{\prime }$ and $G^{\prime \prime }$ values across all frequencies, with $G^{\prime }$ ranging from O(1) to O(10) Pa (Figure 3a). The consistency between these two sets of measurements demonstrates that these gels have the same rheological characteristics, reinforcing the previous hypothesis that BQ is responsible for the gelling observed in Ctrl‐XOS samples. While BQ appears to increase $G^{\prime }$ significantly (over 100‐fold increase compared with the Std‐XOS and chitosan solution), the Ox‐XOS and chitosan hydrogel has a $G^{\prime }$ value of at least O(10) greater than the Ctrl‐XOS or BQ‐XOS hydrogels.

Ctrl‐Lac exhibited a slight increase in $G^{\prime }$ compared to Std‐Lac (less than O(10)). Compared with Std‐Lac, the additional components in the Ctrl‐Lac reaction were catalase and horseradish peroxidase (HRP), both of which were filtered from the reaction solution using a 10 kDa membrane. No differences in chemical composition between Ctrl‐Lac and Lac were detected by HPAEC (Figure 1) or mass spectrometry (Figure S1, Supporting Information), and no oxidase or dehydrogenase activity was observed in Ctrl‐Lac, leaving the origin of this change in $G^{\prime }$ unclear. One possibility is that low‐molecular‐weight impurities in catalase or HRP, which were not completely removed during filtration, may contribute to this effect; however, this hypothesis has yet to be tested. Essentially, enzyme‐oxidized crosslinkers (i.e., Ox‐XOS and Ox‐Lac) yielded significantly higher $G^{\prime }$ values than unmodified carbohydrates (i.e., Ctrl‐XOS, Ctrl‐Lac, Std‐XOS, and Std‐Lac), highlighting the role of enzymatic oxidation in inducing gel‐like behavior.

In LAOS, chitosan hydrogels with Ox‐XOS or Ox‐Lac had $G^{\prime }$ values that remain constant at strains below γ≈40%, with $G^{\prime }$ exceeding G" by one to two orders of magnitude (Figure 3c,d, respectively). However, at higher γ, $G^{\prime }$ began to decrease while $G^{\prime \prime }$ increased and intersected at a critical strain, marking the yield point where the gel transitions from a predominantly elastic (solid‐like) response to a more viscous (liquid‐like) behavior. The yield stress ($\tau_{y}$), a key rheological parameter characterizing the solid‐to‐liquid transition in gels, can be estimated using the relation:

(1) $$\tau_{y}\approx \sqrt{2}\gamma_{c}G_{c},$$

where $\gamma_{c}$ and $G_{c}$ are the strain and modulus at the crossover point, respectively.^[^ ^69^ ^,^ ^70^ ^]^ Using this approach, $\tau_{y}$ is calculated to be 37 and 29 Pa for Ox‐XOS and Ox‐Lac chitosan hydrogels, respectively. Similar values for yield stress are reported for cellulose nanocrystal hydrogels crosslinked with boronate ester bonds for fire prevention materials.^[^ ^73^ ^]^ Applying the same analysis to other formulations, we observed that BQ‐XOS, Ctrl‐XOS, and Ctrl‐Lac gels exhibit similar behavior, with $G^{\prime }>G^{\prime \prime }$ at lower γ and $G^{\prime }<G^{\prime \prime }$ at higher γ. The corresponding $G^{\prime }$ were low compared with gels prepared using Ox‐XOS and Ox‐Lac, where the values were 5.7, 10, and 6.5 Pa respectively for BQ‐XOS, Ctrl‐XOS, and Ctrl‐Lac gels. The higher yield stresses for enzyme‐oxidized crosslinkers (i.e., Ox‐XOS and Ox‐Lac) compared with controls shows there are significant advantages to using the enzyme‐oxidized crosslinkers to make stronger chitosan hydrogels. Meanwhile, Std‐XOS does not exhibit $G^{\prime }>G"$ for any γ, always demonstrating liquid behavior; Lac only displays $G^{\prime }>G^{\prime \prime }$ for a relatively narrow window of γ= 63–156% and therefore has no well‐defined yield point, demonstrating mostly liquid behavior.

**Figure 6:**
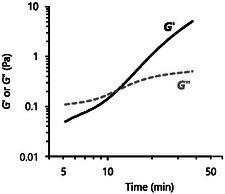
Gelation time for Ox‐XOS and chitosan, measured by the rheometer to be 12 min. Experiments conducted at room temperature and hydrogels contain 138 mM Na_2_HPO_4_, 63 mM acetic acid, and pH ≈ 7.

An experiment was also conducted to quantify the gelation time for Ox‐XOS containing chitosan hydrogels. Gelation time is measured from the initial mixing of Ox‐XOS and chitosan and is defined as the time point at which $G^{\prime }$ surpasses G", indicating that the material's mechanical properties are increasingly governed by solid‐like elastic behavior rather than liquid‐like viscous behavior. The gelation time for a hydrogel made with 40 mM Ox‐XOS and 7.4 mg mL^−1^ chitosan was determined to be 12 min (**Figure** **6**). This result agrees with gelling observations previously discussed, which determined the gelling time for a similar Ox‐XOS and chitosan gel to be around 20 min. A similar measurement was considered for Ox‐Lac, but a precise value was not measured due to the longer gelation times for Ox‐Lac. Combining the data from both of these rheology experiments with previous assessments of gelling as measured by eye, the cutoff for assessing a mixture as a gel versus a solution by eye is a $G^{\prime }$ of $O(10^{-1})$ Pa. These gelation times are similar to those of 10 mg mL^−1^ chitosan solutions crosslinked by 1 mg mL^−1^ genipin, a commonly used bio‐based crosslinker, which were reported as 73–273 min.^[^ ^74^ ^]^

## 4 Conclusions

This study reports the application of carbohydrate oxidoreductases to produce dicarbonyl crosslinkers from underused carbohydrates, which can serve as alternatives to synthetic dicarbonyls such as glutaraldehyde. The enzyme‐oxidized carbohydrates were used to create imine‐crosslinked chitosan hydrogels, confirmed by ^1^H NMR and ATR‐FTIR. The optimal pH range for gel formation was identified to be 7.0–7.2 for both Ox‐XOS and Ox‐Lac. Rheological measurements revealed the impact of enzymatic oxidation, including an increase in the storage modulus ($G^{\prime }$) for chitosan hydrogels with Ox‐XOS of $O(10^{3})$ greater than gels with unmodified XOS and a $G^{\prime \prime }$ of $O(10^{2})$ smaller than its $G^{\prime }$; likewise, Ox‐Lac had a $G^{\prime }$ of $O(10^{2})$ greater than lactose alone and a $G^{\prime \prime }$ of $O(10^{2})$ smaller than $G^{\prime }$. Yield stresses for Ox‐XOS and Ox‐Lac hydrogels were greater than unmodified XOS and lactose. A rheological measurement quantified a gelation time of 12 min for chitosan hydrogels crosslinked with Ox‐XOS.

Future research will explore material properties such as pH and temperature sensitivity, self‐healing ability, and controlled degradability. Parameters including crosslinker concentration, chitosan concentration, pH, and ionic strength will also be varied to evaluate impact on the functionality and performance of the hydrogels. Ultimately, the implications of this work could extend to both high‐value and large‐volume applications, from chitosan hydrogels as biomedical materials for tissue engineering or drug delivery to applications in agriculture, where fully bio‐based materials are especially important.

## Conflict of Interest

The authors declare no conflict of interest.

## Author Contributions

**Owen Mototsune**: conceptualization (equal); methodology (lead); investigation (lead); project administration (lead); visualization (lead); writing—original draft preparation (lead); and writing—review and editing (equal). **Yutong Zhang**: investigation (supporting); and writing—review and editing (supporting). **Durgesh Kavishvar**: investigation by rheological measurements (lead); original draft preparation (supporting); and writing—review and editing (supporting). **Arun Ramchandran**: funding acquisition (supporting); and writing—review and editing (supporting). **Michele Loewen**: funding acquisition (supporting); and writing—review and editing (supporting). **Emma Master**: conceptualization (equal); funding acquisition (lead); project administration (supporting); and writing—review and editing (equal).

## Supporting information

Supplementary Material

## Data Availability

The data that support the findings of this study are available in the supplementary material of this article.
