# Supplementary material for: Enzymatic Oxidation of Carbohydrate Byproducts for Use in Formation of Chitosan Hydrogels
Source: Chembiochem. 2025 Nov 10;26(24):e202500559. doi: 10.1002/cbic.202500559 (PMC12703462; doi:10.1002/cbic.202500559)
Supplement: Supplementary file 1 — Supplementary Material [file CBIC-26-e202500559-s001.pdf]

Supporting Information

Enzymatic Oxidation of Carbohydrate Byproducts for Use in Formation of Chitosan Hydrogels

Authors: Owen Mototsune[1] (0009-0001-2703-4337), Yutong Zhang[2], Durgesh Kavishvar[1] (0000-0002-8243-1503), Arun Ramchandran[1] (0000-0001-9403-5930), Michele C. Loewen[2] (0000-0001-5053-9512), Emma Master[1,3*] (0000-0002-6837-9817)

Affiliations:

1. Department of Chemical Engineering and Applied Chemistry, University of Toronto, 200 College Street, Toronto, ON M5S 3E5, Canada.
2. Department of Chemistry and Biomolecular Sciences, University of Ottawa, 30 Marie Curie, Ottawa, ON K1N 6N5, Canada.
3. Department of Bioproducts and Biosystems, Aalto University, Kemistintie 1, FI-00076, Espoo, Finland.

* corresponding author: emma.master@utoronto.ca

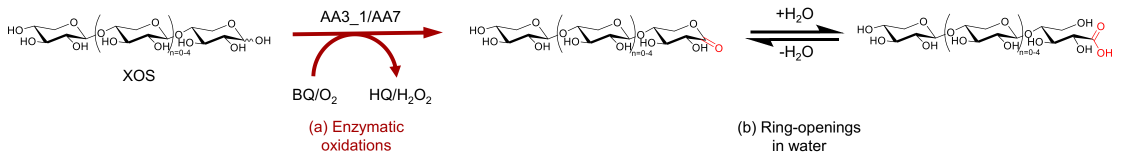

Scheme S 1. Enzymatic oxidations of XOS by AA3_1 or AA7 to produce a C-1 lactone (a) and ring opening in water to convert C-1 lactone to a C-1 acid (b).

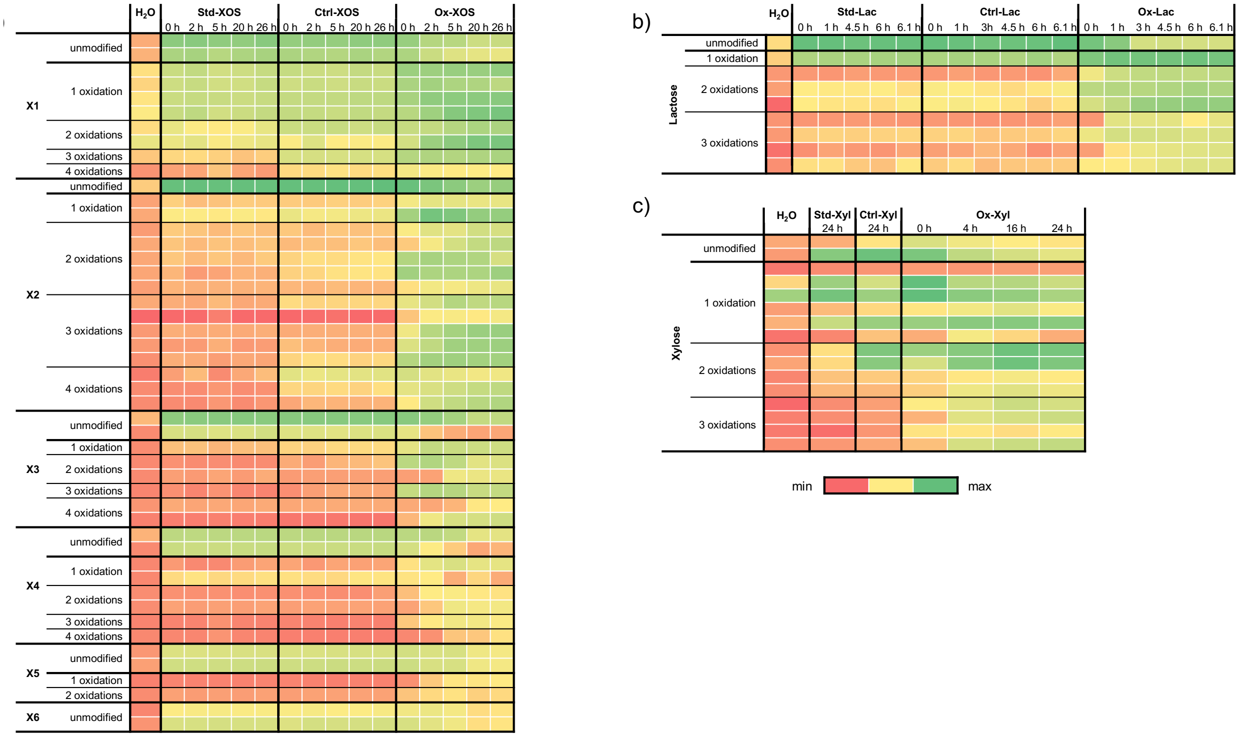

Figure S 1. HILIC-ESI-MS results showing relative depletions of substrates (unmodified xylooligosaccharides (a), xylose (b), and lactose (c)) and relative accumulation of oxidized products for *Ab*PDH1-oxidation of XOS (a) and xylose (b) and *Fgr*GalOx-oxidation of lactose (c). Oxidized carbohydrates are detected to a low but non-zero level in standards and controls, suggesting that some low levels of these compounds are present in the starting materials.

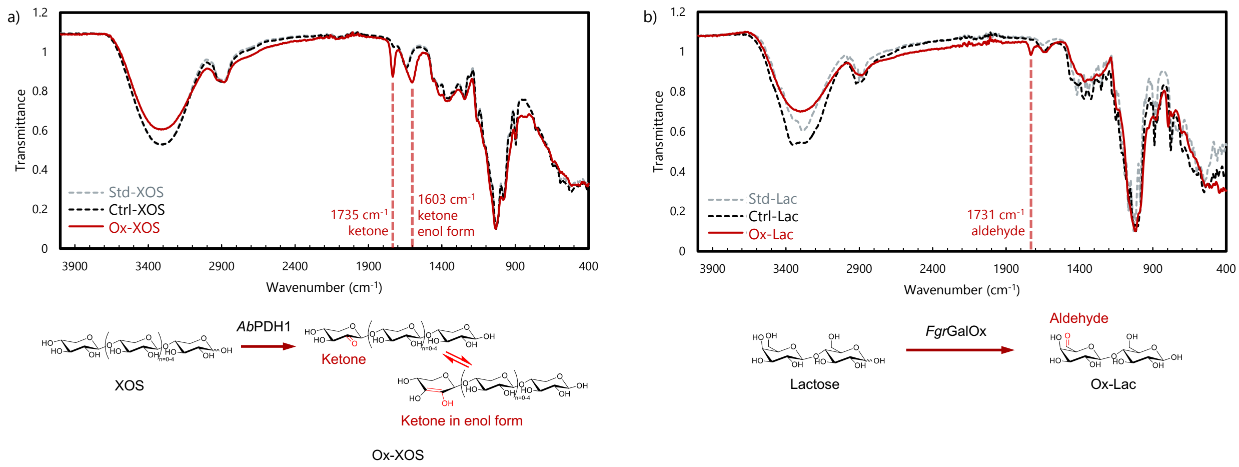

Figure S 2: ATR-FTIR showing introduction of ketone groups by *Ab*PDH1 oxidation of XOS (a) and *Fgr*GalOx-oxidation of Lactose (b).

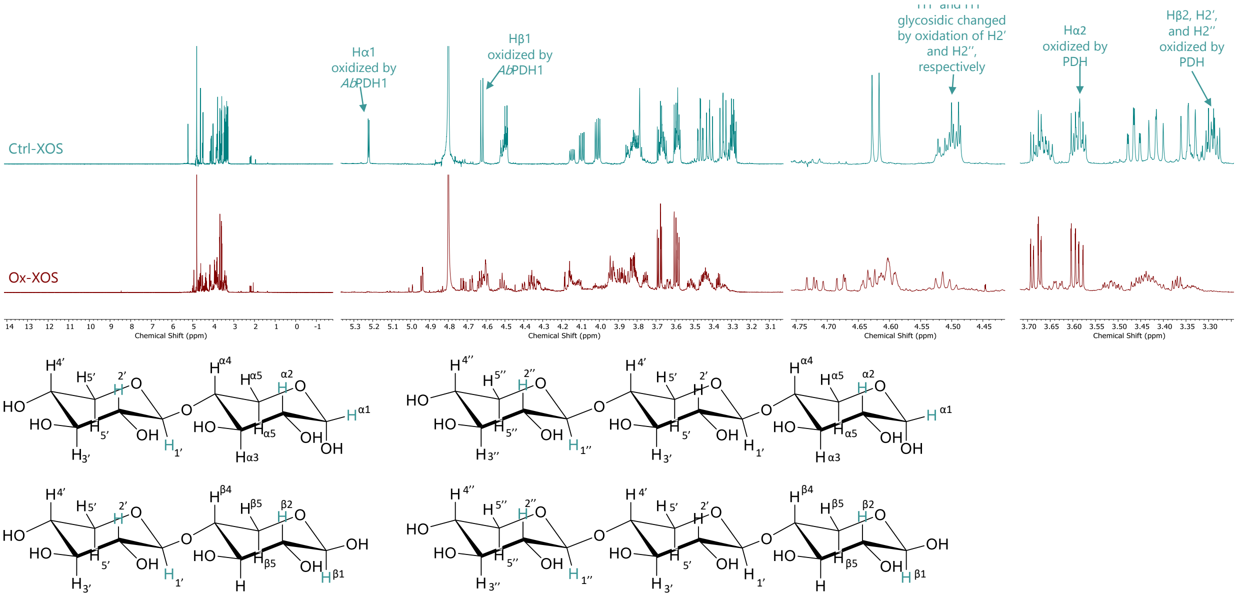

Figure S 3. ^{1}H NMR (700 MHz, d$_2$o) showing oxidation of XOS by *Ab*PDH1, with peaks associated with protons Hα1, Hβ1, Hα2, Hβ2, H2', H2'' reduced in intensity. Detailed 1D and 2D spectra for Ox-XOS and XOS can be found in Figure S 15, Figure S 16, Figure S 17, and Figure S 18.

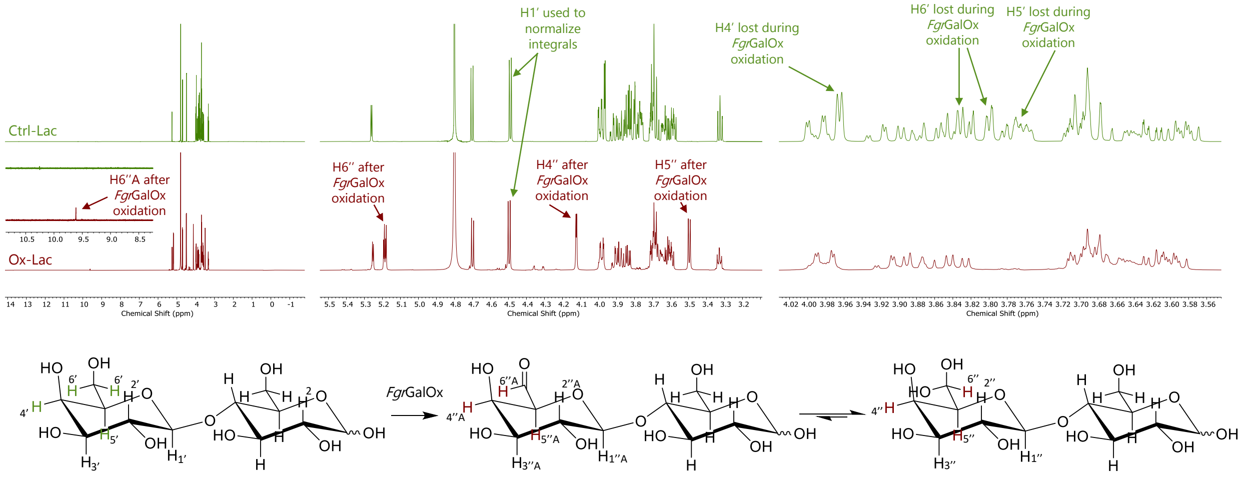

Figure S 4. ^{1}H NMR (700 MHz, d$_2$o) showing oxidation of Lactose by *Fgr*GalOx, with galactosyl protons H4', H5', and H6' converting to H4'', H5'', H6'', and H6''A. Detailed 1D and 2D spectra for Ctrl-Lac and Ox-Lac can be found in Figure S 11, Figure S 12, Figure S 13, and Figure S 14.

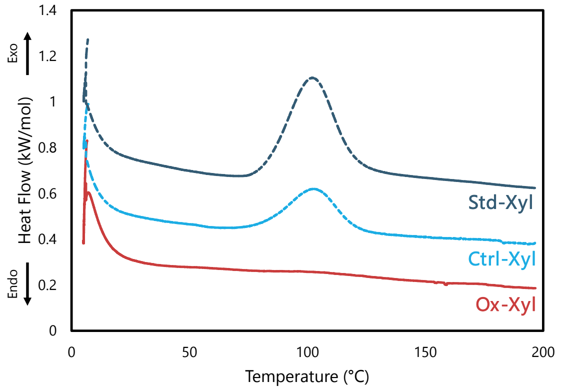

Crosslinker	Temperature (°C) Onset	Peak	Offset	FWHM	Enthalpy of Reaction (kJ/mol)
Std-Xyl	80.6+/-0.4	**101.4+/-0.2**	119.2+/-0.8	23.1+/-1.2	−118.9±11.3
Ctrl-Xyl	80.2	**102.45**	121.2	23.36	−57.6
Ox-Xyl			**no reaction**		

Figure S 5. DSC showing reactivity increase for Ox-Xyl towards HMDA compared to Ctrl-Xyl and Std-Xyl. n=3 for Xyl and Ox-Xyl; n=1 for Ctrl-Xyl.

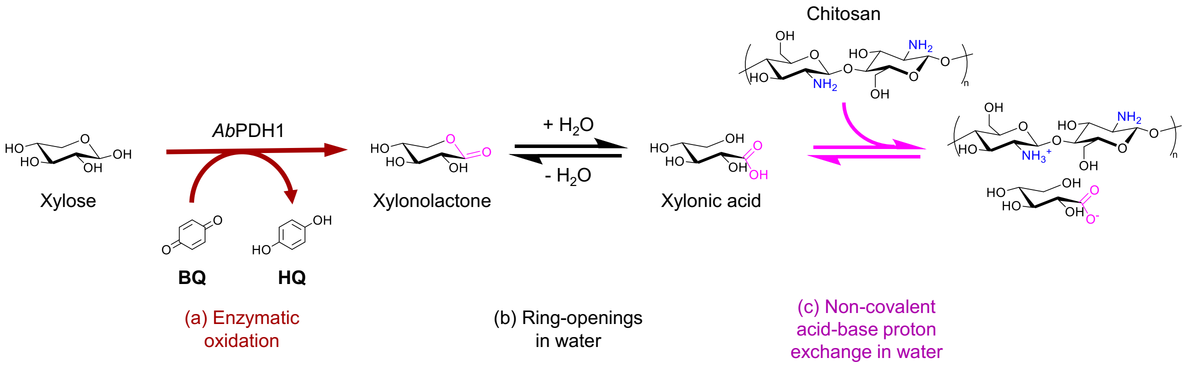

Scheme S 2. Enzymatic oxidations of xylose C-1 hemiacetal by *Ab*PDH1 to produce xylonolactone (a), which hydrates to form a carboxylic acid (xylonic acid) in water (b). Carboxylic acids do not react to form covalent bonds with amines such as chitosan; instead, they donate a proton to be accepted by the amine (c).

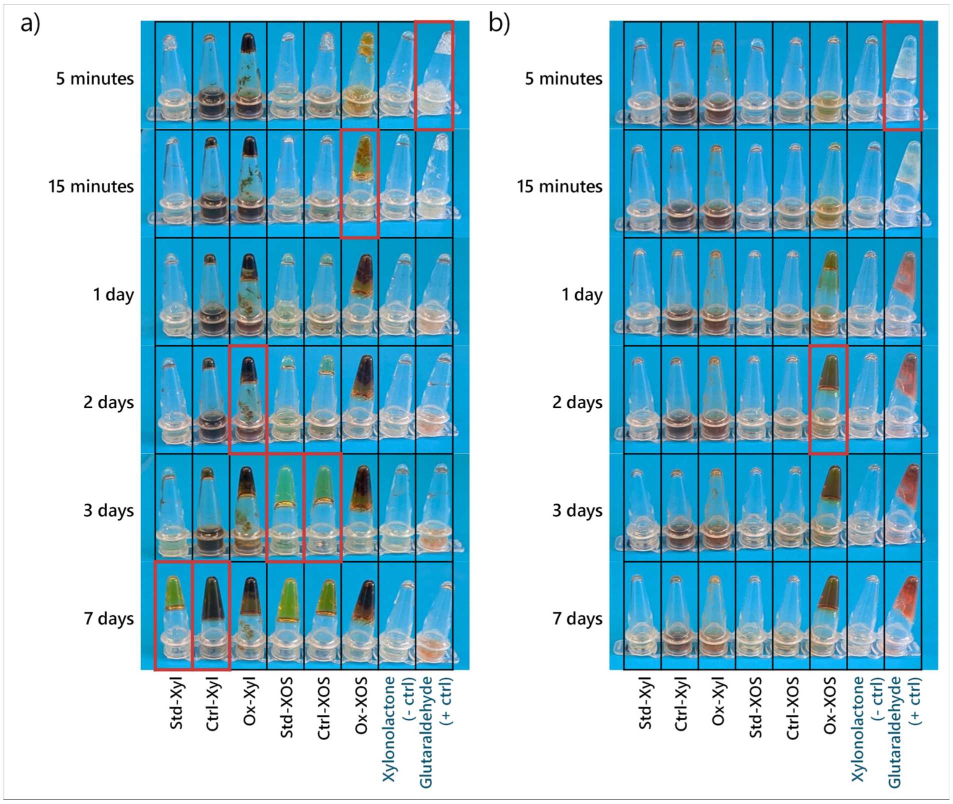

Figure S 6. Photos comparing gel formation ability of Ox-XOS and Ox-Xyl with controls towards 100 mg/mL PAA (a) and 50 mg/mL PAA (b) solutions. Data is compiled into Table 3.

Table S 1. Optimization of pH for chitosan gel formation with Ox-XOS and Ox-Lac. Experiments conducted at room temperature and hydrogels contain 63 mM acetic acid. Italics indicate conditions used for other chitosan gel experiments.

**Na_2HPO_4**		**Gelation time**	
Concentration (mM)	pH	Ox-XOS	Ox-Lac
0	4.35	No gelling*	No gelling*
46	6.28	1.5 h	6 days
69	6.67	1.5 h	2 days
92	6.86	1.5 h	19 h
115	6.99	20 min	19 h
138	*7.06*	*20 min*	*19 h*
161	7.12	20 min	19 h
184	7.21	20 min	19 h
207	7.22	20 min	19 h
230	7.33	1.5 h	19 h
253	7.39	1.5 h	2 days
276	7.43	No gelling*	No gelling*

*no gelling observed over 7-day experiment

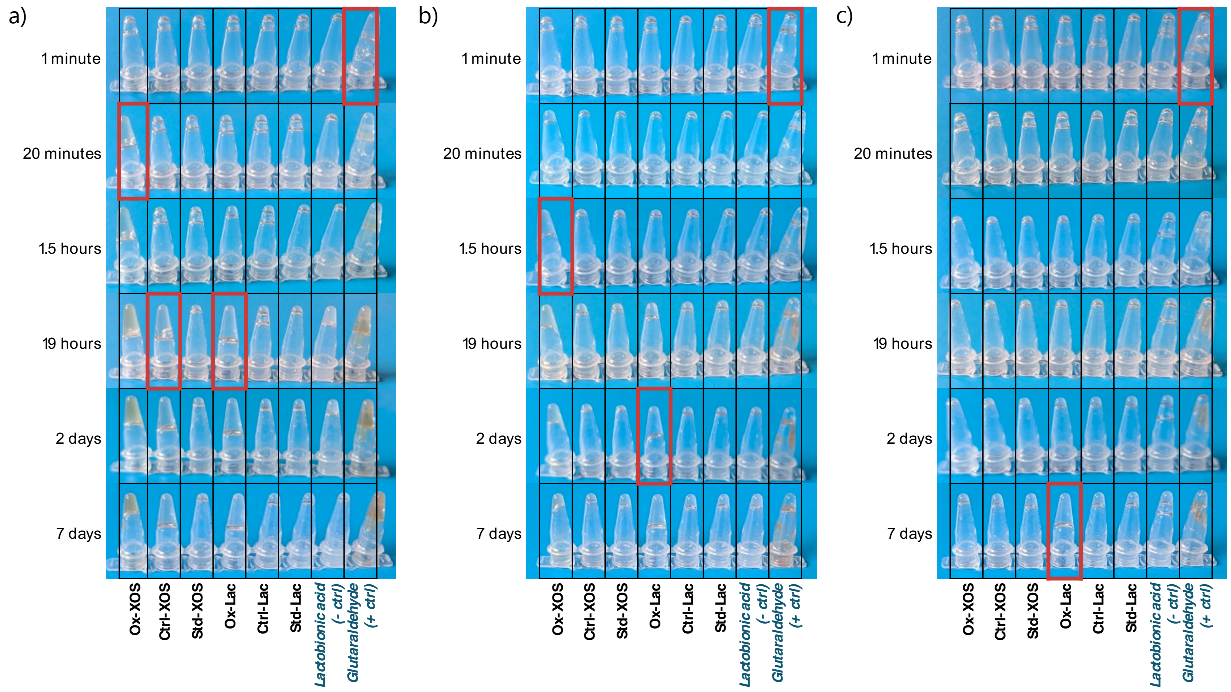

Figure S 7. Photos comparing chitosan hydrogel formation ability of 40 mM (a), 12.5 mM (b), and 4 mM (c) crosslinkers Ox-XOS, and Ox-Lac and controls. Data is compiled into Table 3.

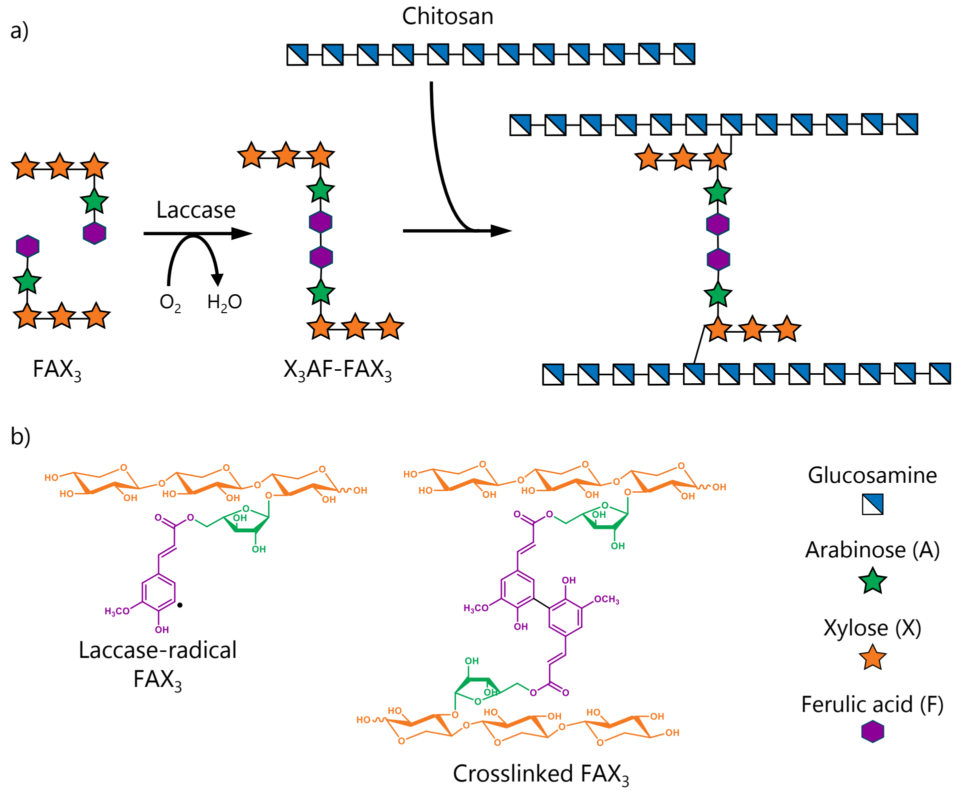

Scheme S 3. Laccase-initiated crosslinking of feruloylated arabinoxylooligosaccharides. Feruloylated arabinoxylooligosaccharides (e.g., FA-AX_3 shown in this diagram) can be oxidized by laccase, which can then recombine into dehydrodimers, which can then act as crosslinkers for chitosan (a). Detailed chemical structures of potential laccase-oxidized feruloylated arabinoxylotriose (FA-AX_3) and crosslinked feruloylated arabinoxylotriose (b).

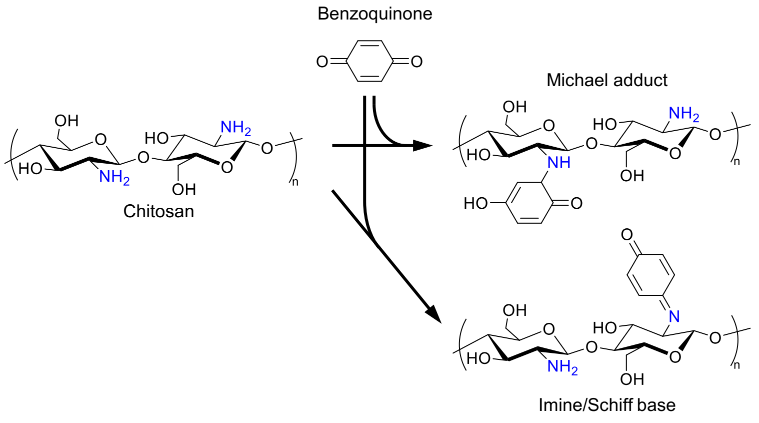

Scheme S 4. Benzoquinone reacting with amine groups in chitosan to form crosslinks through Michael addition or imine bond (Schiff base) formation.

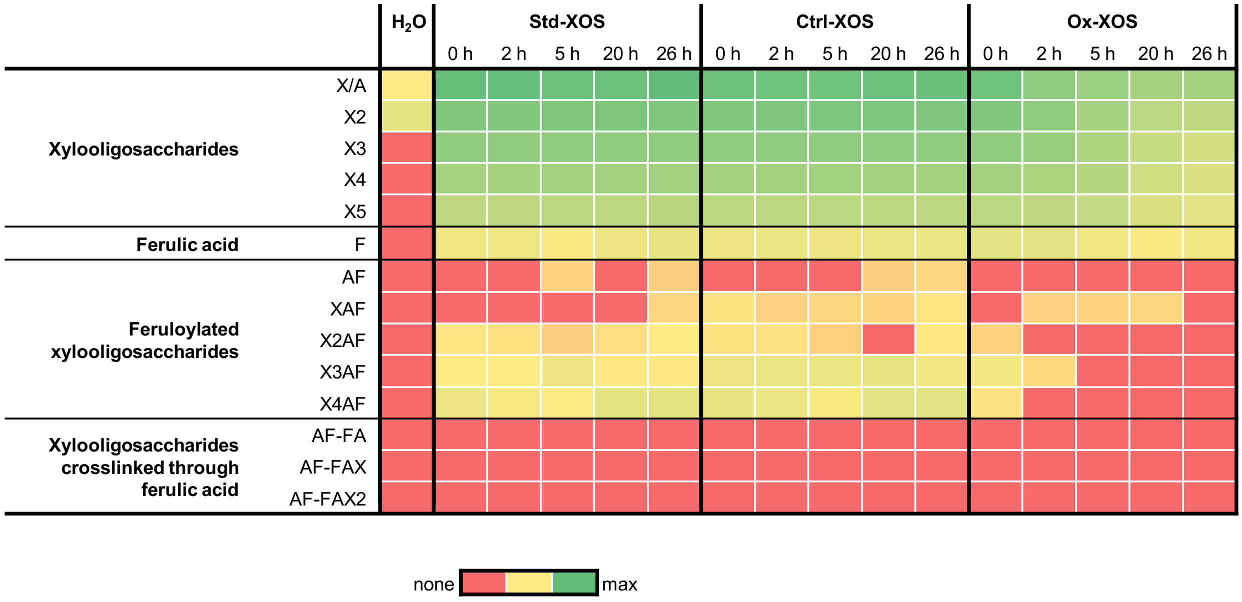

Figure S 8. HILIC-ESI-MS results contrasting relative depletions of unmodified xylooligosaccharides with ferulic acid and feruloylated xylooligosaccharides. While feruloylated xylooligosaccharides are detected in XOS and *Ab*PDH1 is shown to have activity on feruloylated xylooligosaccharides, laccase is not shown to have any activity on feruloylated xylooligosaccharides. In addition, no xylooligosaccharides crosslinked through ferulic acid were detected. Due to m/z constraints of the data acquisition protocol, no larger xylooligosaccharides crosslinked (e.g., AF-FAX$_3$, AF-FAX$_4$) through ferulic acid were detectable.

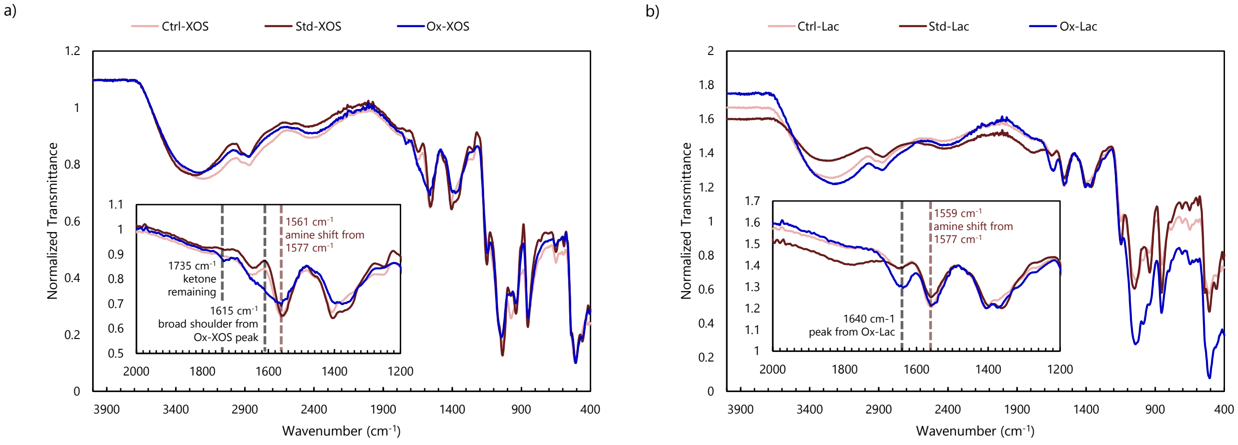

Figure S 9. FTIR showing comparison of reactions of chitosan with controls to reactions with Ox-XOS (a) and Ox-Lac (b).

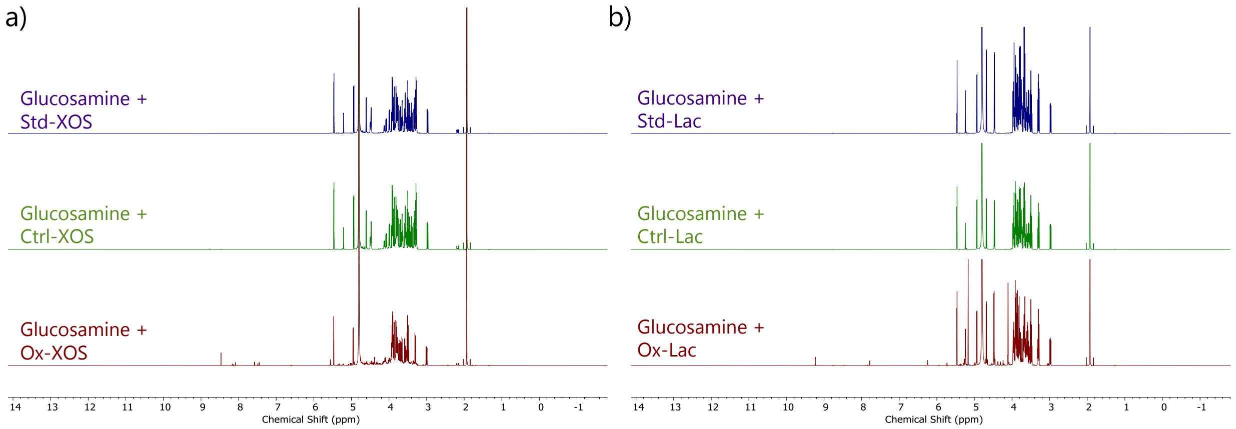

Figure S 10. ^{1}H NMR (700 MHz, d$_2$o) showing imine formation for reactions of glucosamine reactions with Ox-XOS and Ox-Lac (b) but not for reactions with controls and standards with no reaction observed. All samples analyzed after 48 h reactions.

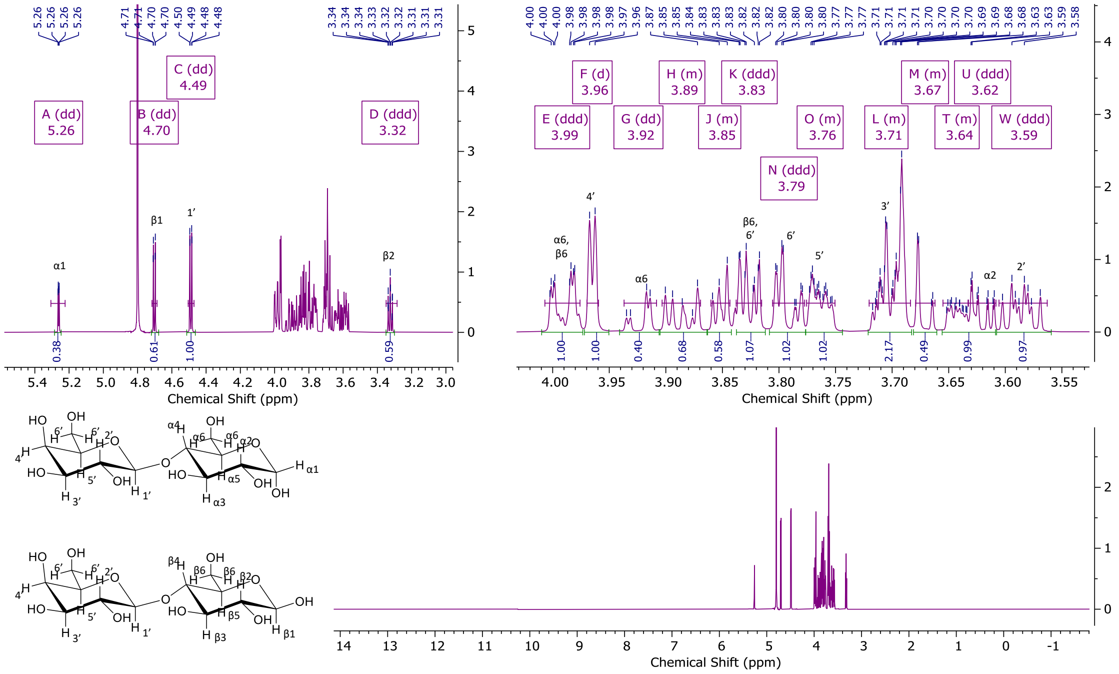

Figure S 11. ^{1}H NMR spectra of Ctrl-Lac. ^{1}H NMR (700 MHz, d_2o) δ 5.26 (dd, J = 3.9, 0.7 Hz, 0.4H), 4.70 (d, J = 8.0 Hz, 0.6H), 4.54 – 4.46 (m, 1.0H), 3.99 (ddd, J = 12.3, 2.3, 0.8 Hz, 1.0H), 3.96 (d, J = 3.4 Hz, 1.0H), 3.92 (dd, J = 12.3, 2.3 Hz, 0.4H), 3.90 – 3.87 (m), 3.86 – 3.84 (m), 3.83 (ddd, J = 8.2, 3.4, 0.7 Hz, 1.6H), 3.79 (ddd, J = 11.8, 3.9, 0.9 Hz, 1.0H), 3.78 – 3.75 (m, 1.0H), 3.72 – 3.68 (m), 3.68 – 3.66 (m), 3.66 – 3.63 (m), 3.62 (ddd, J = 9.9, 3.8, 0.7 Hz), 3.59 (ddd, J = 9.8, 7.7, 5.6 Hz, 1H), 3.32 (ddd, J = 8.9, 8.0, 0.8 Hz, 0.6). Because lactose exists as α and β anomers, the total signal from both anomers was sum to 1.00. Because protons on the galactosyl subunit are not influenced in a measurable way by the anomeric protons, the signals of protons on the galactosyl subunit, e.g., H1’, do not split into separate signals and have integrals equal to ~1.00.

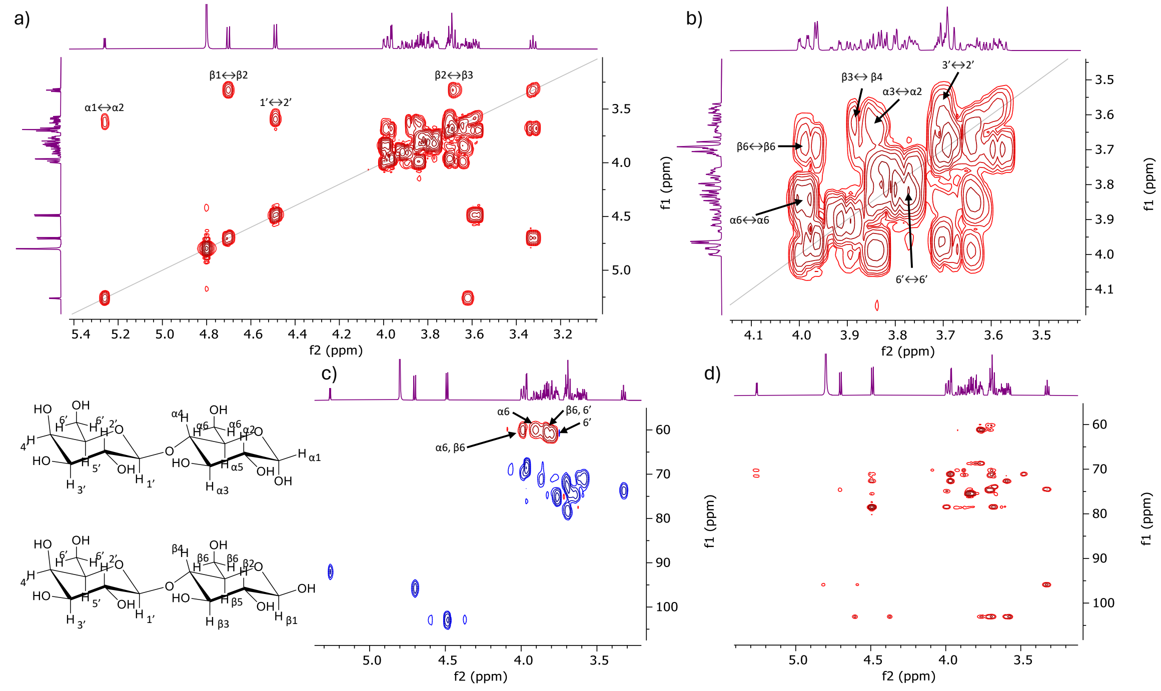

Figure S 12. 2D NMR (700 MHz, d_2o) spectra of Ctrl-Lac. 2D COSY-45 (a, b), 2D ^{1}H/^{13}C multiplicity-edited HSQC (c), 2D ^{1}H/^{13}C HMBC (d) spectra.

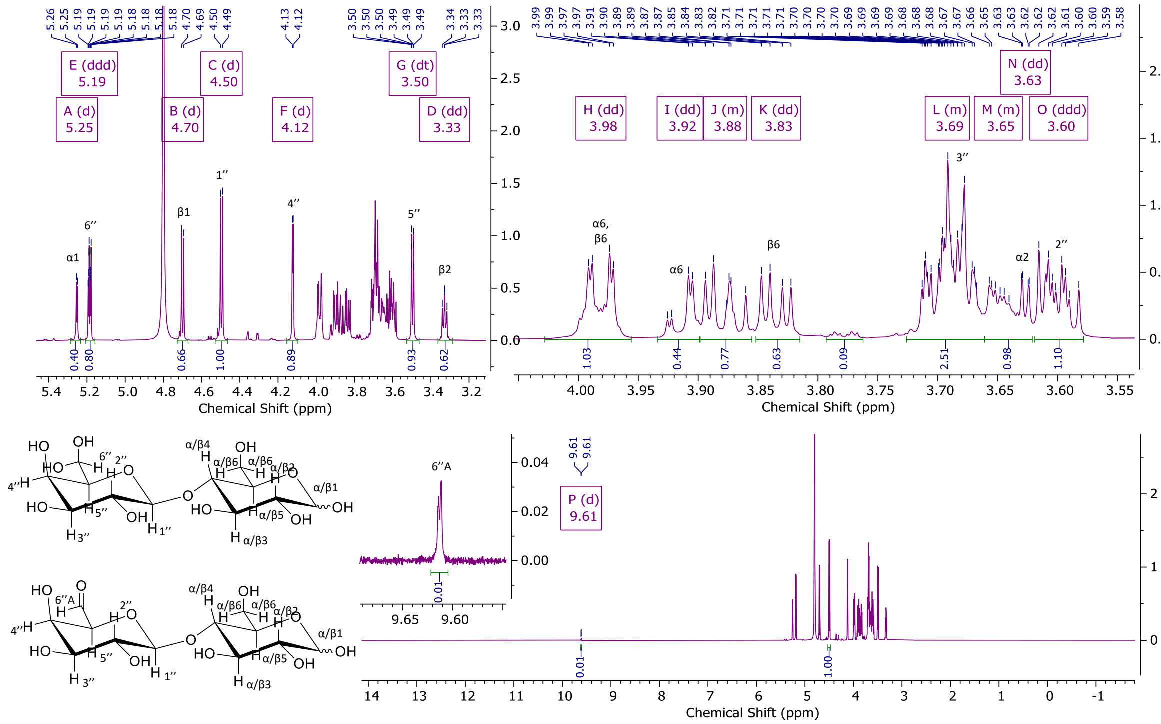

Figure S 13. ^{1}H NMR spectra of Ox-Lac. ^{1}H NMR (700 MHz, d$_2$o) δ 9.61 (d, J = 1.8 Hz, 1H), 5.25 (d, J = 3.8 Hz, 0.4H), 5.19 (ddd, J = 7.4, 3.2, 0.6 Hz, 0.8H), 4.70 (d, J = 8.2 Hz, 0.6H), 4.50 (d, J = 7.9 Hz, 1H), 4.12 (d, J = 3.0 Hz, 0.8H), 3.98 (dd, J = 12.3, 2.3 Hz, 1H), 3.92 (dd, J = 12.3, 2.5 Hz, 0.4H), 3.90 – 3.86 (m), 3.83 (dd, J = 12.2, 5.0 Hz, 0.6H), 3.73 – 3.67 (m), 3.66 – 3.64 (m), 3.63 (dd, J = 3.8, 0.6 Hz, 0.4H), 3.60 (ddd, J = 9.9, 7.8, 5.6 Hz), 3.50 (dt, J = 7.4, 0.9 Hz, 0.8H), 3.33 (dd, J = 9.4, 7.9 Hz, 0.8H).

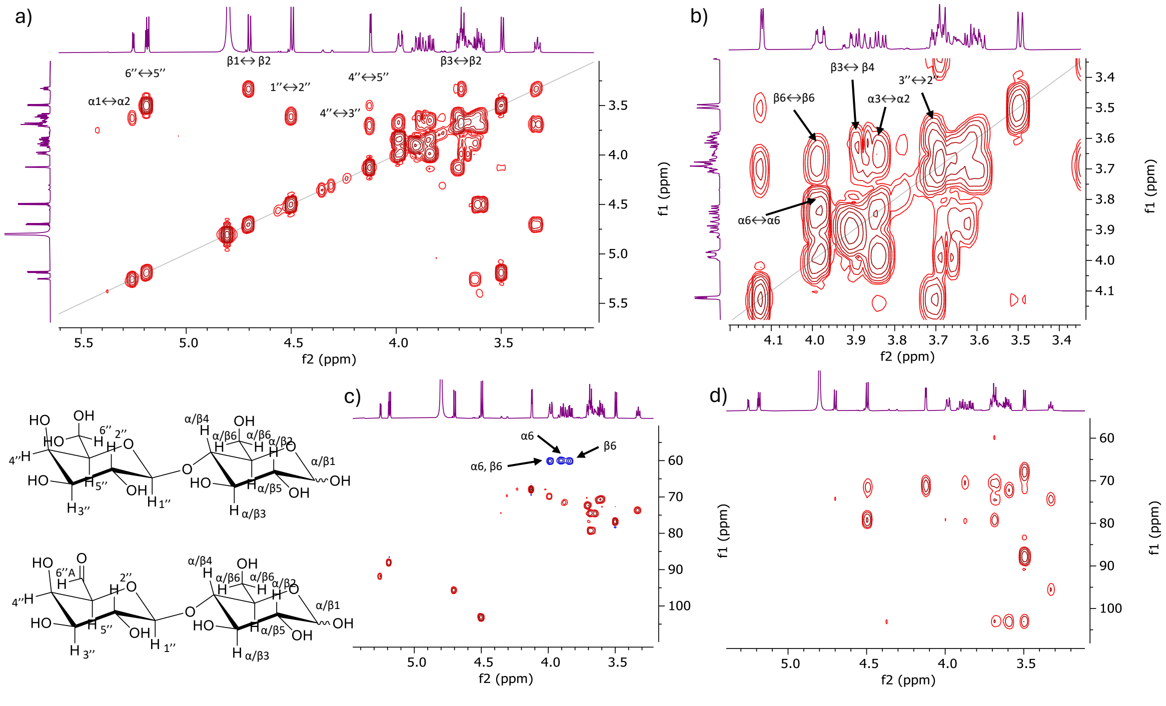

Figure S 14. 2D NMR (700 MHz, d$_2$o) spectra of Ox-Lac. 2D COSY-45 (a, b), 2D ^{1}H/^{13}C multiplicity-edited HSQC (c), 2D ^{1}H/^{13}C HMBC (d) spectra.

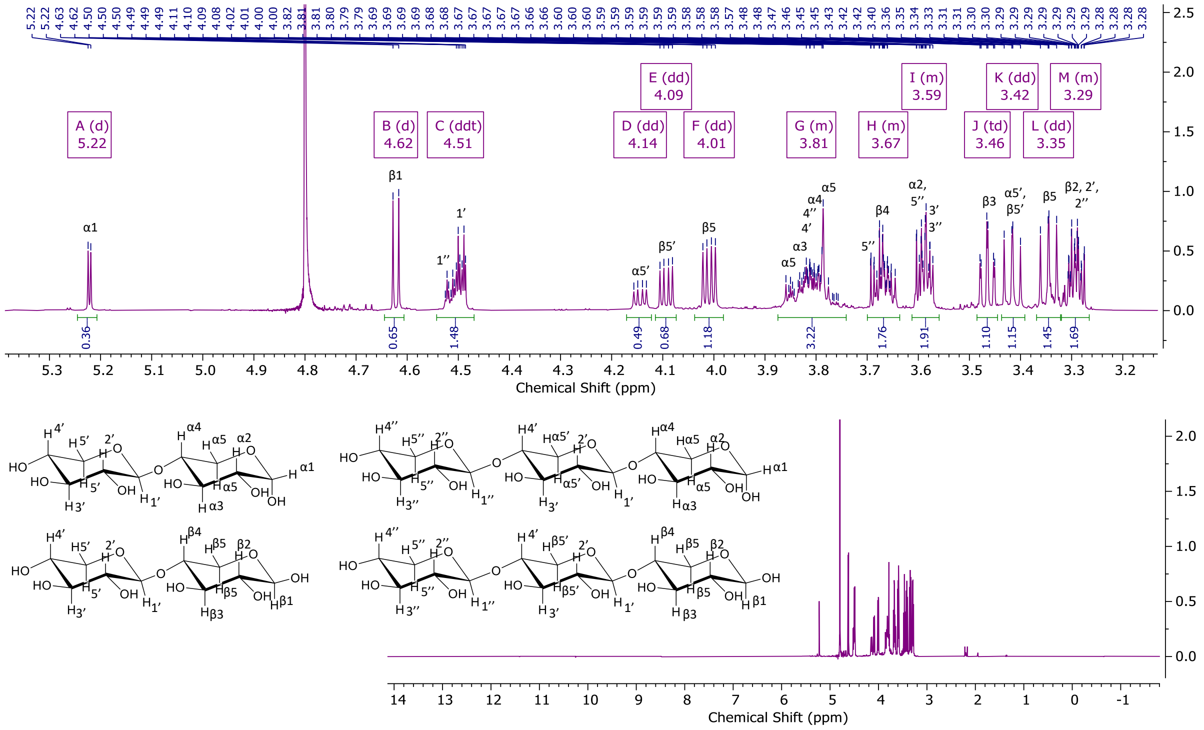

Figure S 15. ^{1}H NMR spectra of Ctrl-XOS. ^{1}H NMR (700 MHz, d_2o) δ 5.22 (d, J = 3.7 Hz, 0.4H), 4.62 (d, J = 7.8 Hz, 0.6H), 4.51 (ddt, J = 15.3, 7.8, 2.3 Hz, 1.5H), 4.14 (dd, J = 11.8, 5.4 Hz, 0.4H), 4.09 (dd, J = 11.7, 5.4 Hz, 0.6H), 4.01 (dd, J = 11.6, 5.5 Hz, 0.6H), 3.87 – 3.75 (m), 3.70 – 3.64 (m), 3.61 – 3.57 (m), 3.46 (td, J = 9.3, 1.6 Hz, 0.6H), 3.42 (dd, J = 11.8, 10.5 Hz), 3.35 (dd, J = 11.6, 10.6 Hz, 0.6H), 3.31 – 3.27 (m).

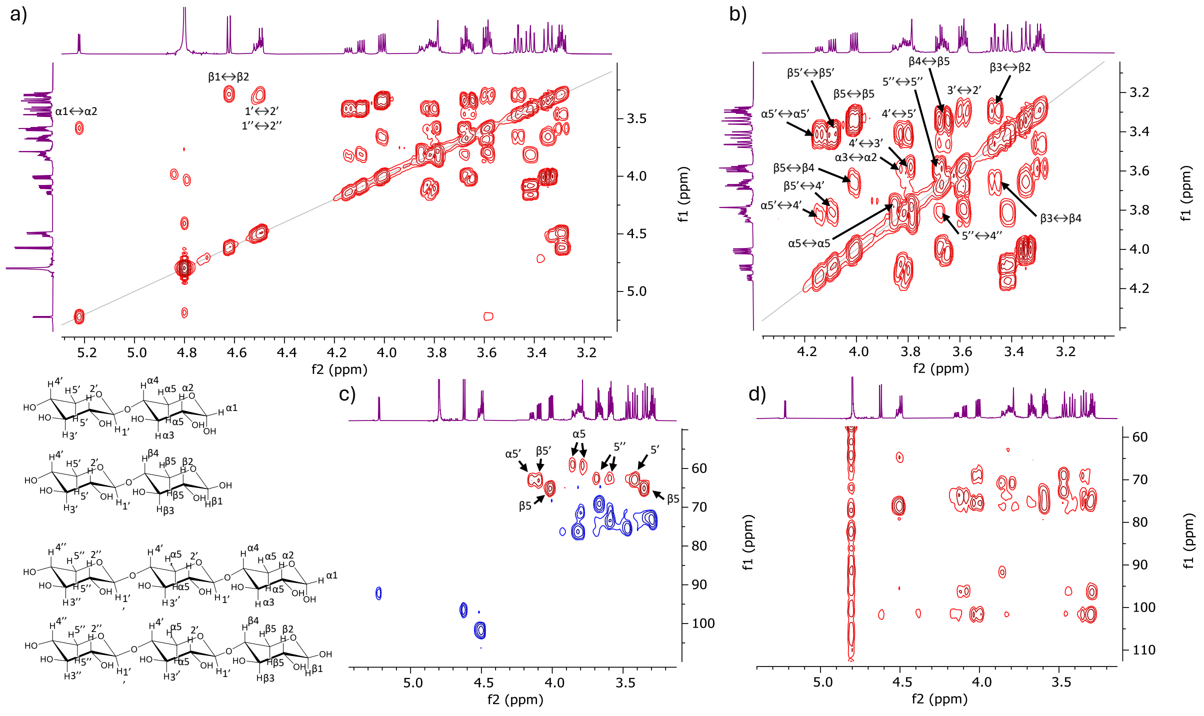

Figure S 16. 2D NMR (700 MHz, d_2o) spectra Ctrl-XOS. 2D COSY-45 (a, b), 2D ^{1}H/^{13}C multiplicity-edited HSQC (c), 2D ^{1}H/^{13}C HMBC (d) spectra.

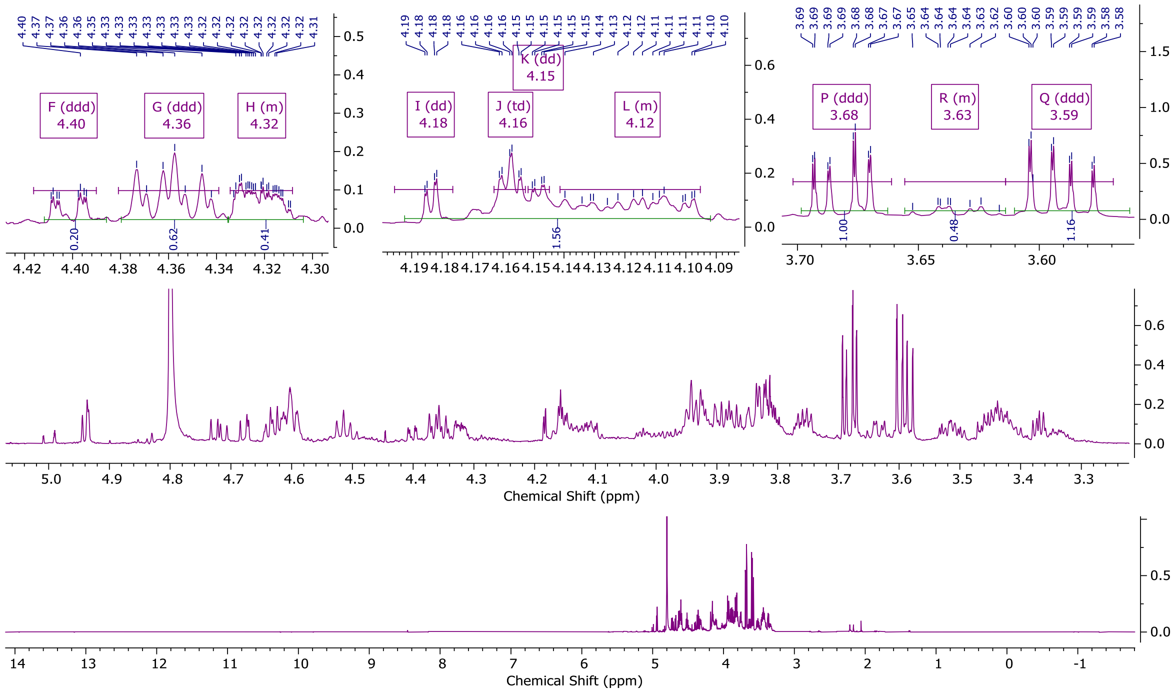

Figure S 17. ^{1}H NMR spectra of Ox-XOS. ^{1}H NMR (700 MHz, d_2o) δ 4.95 – 4.93 (m), 4.72 (dd, J = 10.5, 7.6 Hz), 4.69 – 4.66 (m), 4.65 – 4.58 (m), 4.51 (q, J = 7.7 Hz), 4.40 (ddd, J = 7.9, 1.7, 0.6 Hz), 4.36 (ddd, J = 10.7, 7.7, 2.8 Hz), 4.33 – 4.31 (m), 4.18 (dd, J = 2.2, 0.5 Hz), 4.16 (td, J = 2.2, 0.6 Hz), 4.15 (dd, J = 2.1, 0.6 Hz), 4.14 – 4.10 (m), 4.04 – 3.96 (m), 3.96 – 3.79 (m), 3.78 – 3.74 (m), 3.68 (ddd, J = 11.8, 4.4, 0.6 Hz), 3.66 – 3.61 (m), 3.59 (ddd, J = 11.8, 6.5, 0.6 Hz), 3.55 – 3.49 (m), 3.48 – 3.38 (m), 3.39 – 3.30 (m).

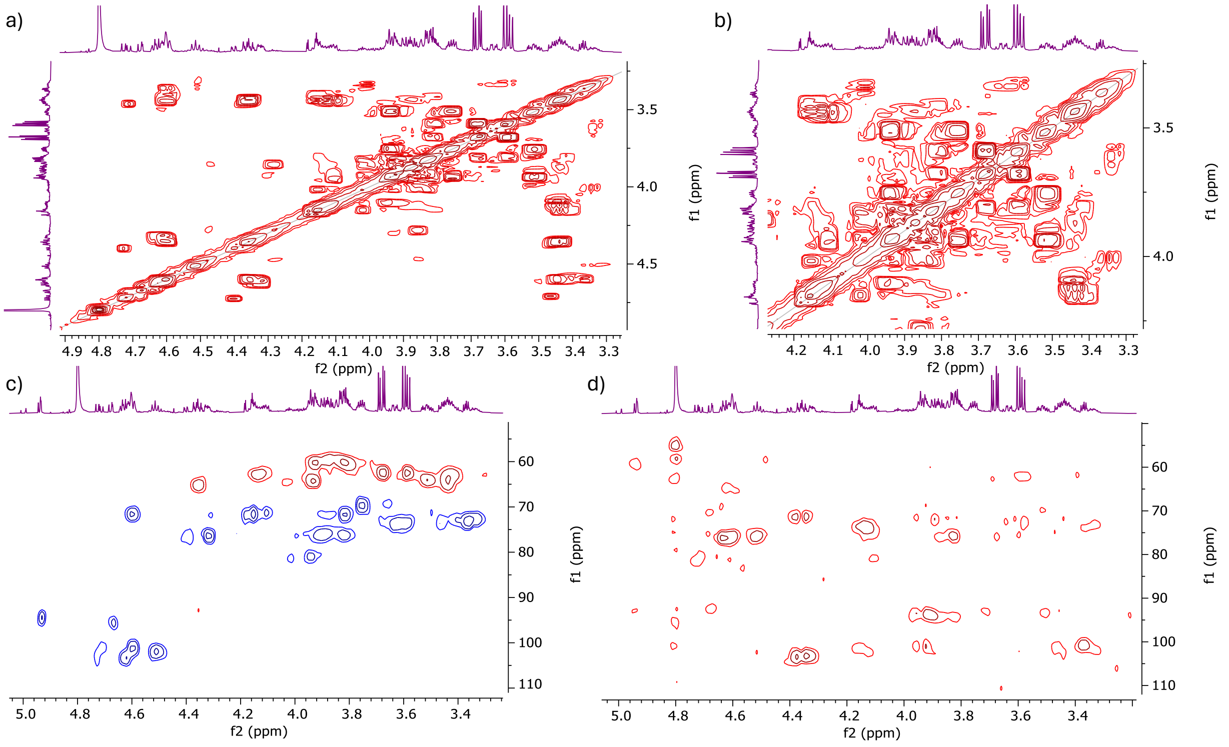

Figure S 18. 2D NMR (700 MHz, d_2o) spectra Ox-XOS. 2D COSY-45 (a, b), 2D ^{1}H/^{13}C multiplicity-edited HSQC (c), 2D ^{1}H/^{13}C HMBC (d) spectra.

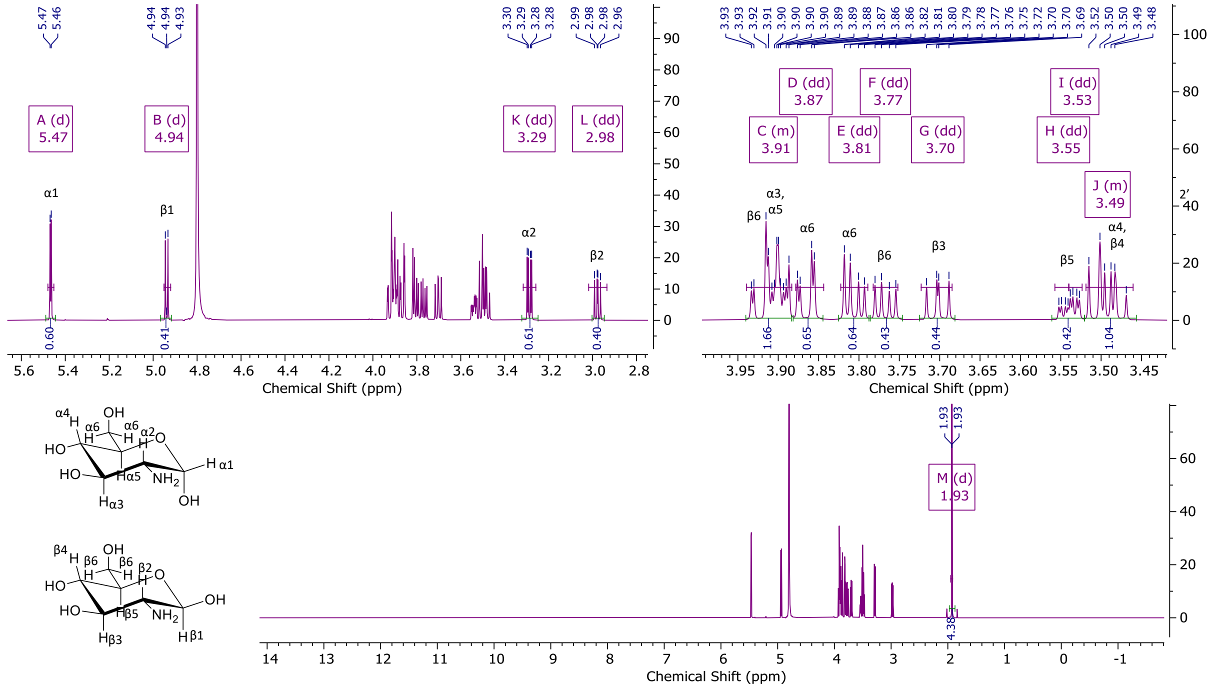

Figure S 19. ^{1}H NMR spectra of GluN. ^{1}H NMR (700 MHz, d_2o) δ 5.47 (d, J = 3.6 Hz, 0.6H), 4.94 (d, J = 8.4 Hz, 0.4H), 3.94 – 3.88 (m), 3.87 (dd, J = 12.3, 2.3 Hz, 0.6H), 3.81 (dd, J = 12.3, 5.1 Hz, 0.6H), 3.77 (dd, J = 12.4, 5.6 Hz, 0.4H), 3.70 (dd, J = 10.6, 8.8 Hz, 0.4H), 3.55 (dd, J = 5.7, 2.3 Hz, 0.4H), 3.53 (dd, J = 5.6, 2.2 Hz), 3.52 – 3.46 (m, 1.0H), 3.29 (dd, J = 10.6, 3.6 Hz, 0.6H), 2.98 (dd, J = 10.6, 8.4 Hz, 0.4H). Peak at δ 1.93 (d, J = 0.6 Hz) is due to acetic acid used in sample preparation.

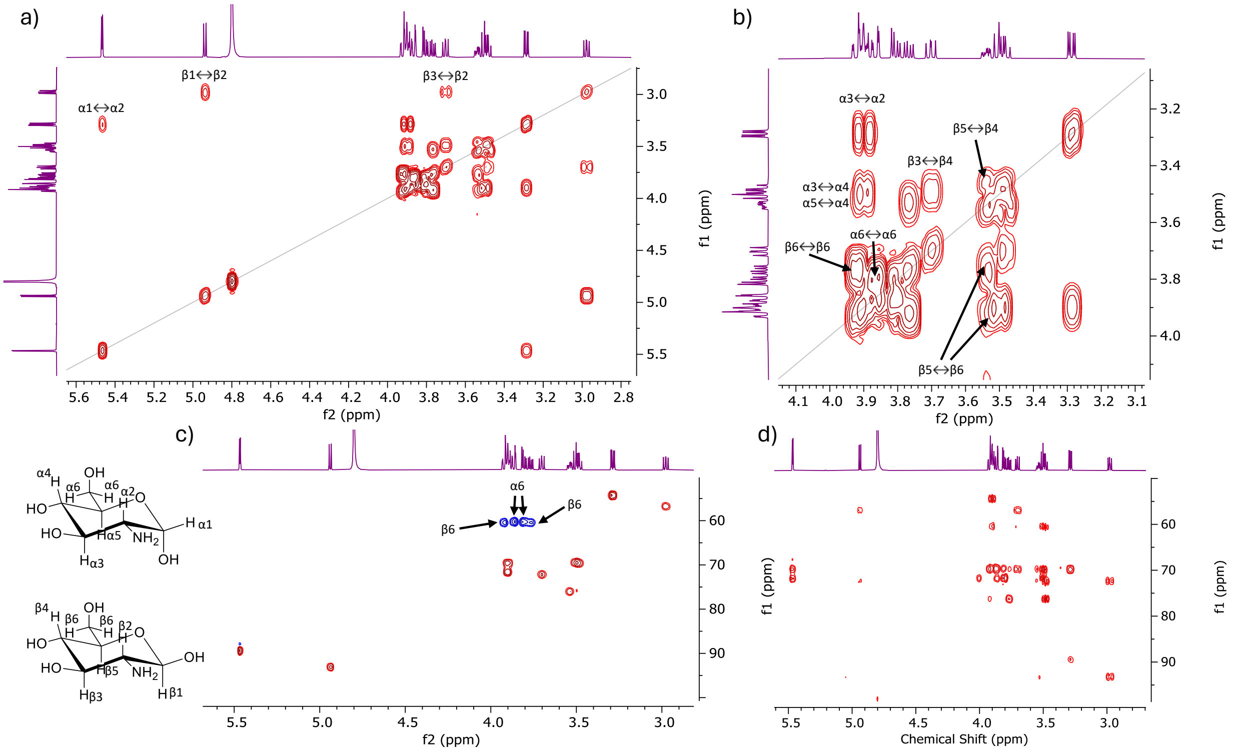

Figure S 20. 2D NMR (700 MHz, d_2o) spectra GluN. 2D COSY-45 (a, b), 2D ^{1}H/^{13}C multiplicity-edited HSQC (c), 2D ^{1}H/^{13}C HMBC (d) spectra.
